# Multi‐Omics Reveals Early Pregnancy Placental Dysfunction Associated With Preterm and Term Preeclampsia

**DOI:** 10.1002/mco2.70784

**Published:** 2026-05-26

**Authors:** Ellen Menkhorst, Guannan Yang, Yimiao Yu, Leilani L. Santos, Wei Zhou, Argyro Syngelaki, Swati Varshney, Nicholas A. Williamson, Kaori Koga, Daniel Lorber Rolnik, Fabricio da Silva Costa, Kypros Nicolaides, Kim‐Anh Lê Cao, Evdokia Dimitriadis

**Affiliations:** ^1^ Department of Obstetrics Gynaecology and Newborn Health The University of Melbourne Parkville Victoria Australia; ^2^ Gynaecology Research Centre Royal Women's Hospital Parkville Victoria Australia; ^3^ Melbourne Integrative Genomics School of Mathematics and Statistics The University of Melbourne Parkville Victoria Australia; ^4^ Fetal Medicine Foundation London UK; ^5^ Harris Birthright Research Centre for Fetal Medicine King's College Hospital London UK; ^6^ Melbourne Mass Spectrometry and Proteomics Facility Bio21 Molecular Science & Biotechnology Institute The University of Melbourne Melbourne Victoria Australia; ^7^ Department of Obstetrics and Gynecology The University of Tokyo Tokyo Japan; ^8^ Department of Obstetrics and Gynecology Reproductive Medicine Chiba University Chiba Japan; ^9^ Department of Obstetrics and Gynaecology Monash University Melbourne Victoria Australia; ^10^ Women's and Newborn Monash Health Melbourne Victoria Australia; ^11^ Maternal Fetal Medicine Unit Gold Coast University Hospital Gold Coast Queensland Australia; ^12^ School of Medicine and Dentistry Griffith University Gold Coast Queensland Australia

**Keywords:** chorionic villus samples, melanophilin, multi‐omics, placenta, preeclampsia

## Abstract

Preeclampsia, a severe pregnancy‐induced disorder unique to humans, affects ∼2%–8% of pregnancies globally. Strong evidence supports placental dysfunction as central to preeclampsia; however, there is inadequate understanding of the precise pathogenesis of preeclampsia. In this study, we present a comprehensive multi‐omics analysis of early pregnancy placental biopsies (chorionic villus samples) from pregnancies that later developed preterm/term preeclampsia, compared to normotensive controls. Using an integrative multivariate approach, we uncovered distinct molecular signatures associated with preeclampsia. Preterm preeclampsia was strongly associated with dysregulated lipoprotein metabolism, while term preeclampsia exhibited alterations in inflammatory pathways, Notch/Kit signaling, and ribosome assembly. These results challenge the prevailing notion that term preeclampsia is unrelated to early placental pregnancy dysfunction. To validate our findings, we focused on melanophilin, a gene downregulated in the early pregnancy placenta of term preeclampsia. Melanophilin expression was reduced during cytotrophoblast syncytialization; however, excessive loss disrupted syncytiotrophoblast function, triggering the production of factors known to drive preeclampsia. Our study provides critical insights into the early pregnancy aberrations underlying preterm and term preeclampsia, paving the way for the development of predictive biomarkers and targeted preventative treatments. This work represents a significant step toward unraveling the complex etiology of preeclampsia and improving maternal and perinatal health outcomes.

## Graphical abstract

Preeclampsia, a severe pregnancy‐induced disorder unique to humans, affects ∼5% of pregnancies globally. To understand the pathogenesis of preeclampsia, this study performed a comprehensive multi‐omics analysis of early pregnancy placental biopsies (chorionic villus samples) from pregnancies that later developed preterm/term preeclampsia, compared to normotensive controls. Using an integrative multivariate approach, we uncovered distinct molecular signatures associated with preterm and term preeclampsia, including melanophilin, whose loss increased syncytiotrophoblast production of known drivers of preeclampsia, including sFLT1. Created in BioRender. Menkhorst, E. (2026). Permission: https://BioRender.com/voktssv.

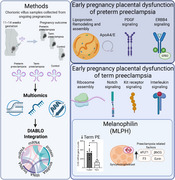

## 1 Introduction

Preeclampsia, a severe pregnancy‐induced disorder unique to humans [1], affects 2%–8% of pregnancies globally [2], resulting in over four million cases annually [3, 4]. Preeclampsia leads to the deaths of 46,000 women and 500,000 babies each year [5] and increases long‐term chronic disease risk in both mothers and children [4, 6].

Clinically, preeclampsia manifests as a complex multi‐system disease characterized by the development of new‐onset hypertension after 20 weeks’ gestation, accompanied by at least one associated complication such as proteinuria, maternal organ dysfunction, or placental dysfunction [1, 6, 7]. Strong evidence supports placental dysfunction as central to preeclampsia, because the condition arises only in the presence of a placenta or shortly after its delivery (post‐partum preeclampsia) [1]. However, we have an inadequate understanding of the precise pathogenesis of preeclampsia, leading to very few predictive biomarkers or targeted preventative treatments.

The timing of preeclampsia onset, which can be classed as preterm (delivery < 37 weeks’ gestation) and term (delivery ≥ 37 weeks’ gestation), is thought to reflect underlying pathophysiological differences [1]. Regardless of the initial trigger, in both preterm and term preeclampsia, syncytiotrophoblast stress is thought to lead to the placenta abnormally releasing pro‐inflammatory cytokines and anti‐angiogenic factors into the maternal circulation, driving maternal endothelial dysfunction and the symptoms of preeclampsia [1]. Preterm preeclampsia is thought to result from abnormal placentation during early pregnancy, including poor spiral artery remodeling, which results in placental ischemia [8, 9] and syncytiotrophoblast stress [1]. Conversely, abnormal placental histopathological findings are uncommon in term preeclampsia [10, 11], leading to the conclusion that the syncytiotrophoblast stress of term preeclampsia is initiated later in pregnancy due to compression of placental chorionic villus or premature placental aging [12].

The belief that the term preeclampsia is not associated with early pregnancy placental dysfunction [13] has never been experimentally tested. Moreover, the specific early pregnancy placental molecular changes associated with preterm and term preeclampsia are largely unknown, partly due to the challenges in obtaining suitable samples. Elucidating these early pregnancy placental molecular changes is crucial for developing biomarkers to predict risk and therapeutic interventions to improve outcomes in preeclampsia.

To address this knowledge gap, we conducted a comprehensive omics analysis of early pregnancy placental biopsies (chorionic villus samples [CVS]) collected at 11–14 weeks’ gestation from pregnancies that later developed preterm preeclampsia, term preeclampsia, or remained normotensive. Recognizing that a single omics approach is insufficient to fully characterize complex disease states [14], we assessed multiple regulatory levels (mRNA, noncoding RNA, and protein) to gain a holistic understanding of the molecular mechanisms driving preeclampsia [15]. Our data revealed key pathways dysregulated in the early pregnancy placenta of pregnancies destined to develop preterm (lipoprotein metabolism) or term (inflammation, Notch and Kit signaling, ribosome assembly) preeclampsia. We coupled this multi‐omics analysis with functional studies in human trophoblast to reveal melanophilin (MLPH) as a key factor associated with cell fusion during syncytialization, and which is significantly downregulated in placental villus from term preeclampsia. This work represents a significant step toward unraveling the complex etiology of preeclampsia and improving maternal and perinatal health outcomes.

## 2 Results

### 2.1 Resolving the Key Pathways Dysregulated in Preeclampsia Using Multi‐Omics

**FIGURE 1:**
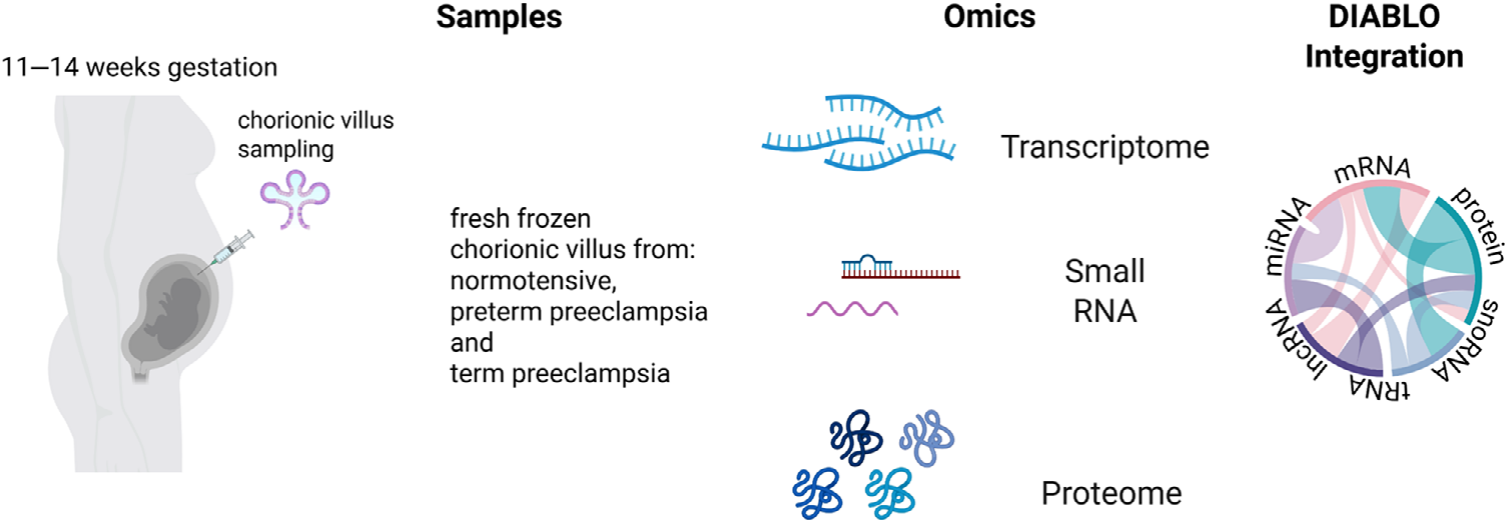
Schematic diagram of protocol for multi‐omics analysis. Normotensive control, *n* = 6; preterm preeclampsia, *n* = 4; term preeclampsia, *n* = 4. Figure created in BioRender. Menkhorst, E. (2025). Permission: https://BioRender.com/u76e841.

To identify the key pathogenic molecules dysregulated in the early pregnancy placenta of pregnancies that subsequently develop preterm and term preeclampsia, we performed a comprehensive multi‐omics screen using CVS obtained between 11 and 14 weeks’ gestation (Figure 1 and Table 1). We employed DIABLO (Data Integration Analysis for Biomarker discovery using Latent cOmponents) [15], a supervised multivariate method, to integrate and analyze the multi‐omics data. This approach identified highly correlated omics variables and generated linear combinations (“components”) capable of discriminating between control, preterm preeclampsia, and term preeclampsia groups.

**TABLE 1:** Clinical characteristics of chorionic villus samples used in multi‐omics.

	Normotensive control	Preterm preeclampsia	Term preeclampsia	
Sample size (*n*)	6	4	4	
Maternal age (years)	35.78 ± 2.484 (27, 42)	37.28 ± 3.140 (30, 44)	34.05 ± 3.994 (25, 44)	F_2, 11_ 0.2318 *p* = 0.79
Maternal BMI	25.03 ± 2.582 (19.1, 36.1)	27.68 ± 4.541 (18.1, 38.5)	27.80 ± 2.342 (24.2, 34.2)	F_2, 11_ 0.2690 *p* = 0.77
Gestational age CVS collection (weeks)	12.73 ± 0.3556 (11.7, 14.1)	12.43 ± 0.1377 (12.1, 12.7)	12.78 ± 0.4802 (11.6, 13.7)	F_2, 11_ 0.2479 *p* = 0.78
Gestational age at delivery (weeks)	40.33 ± 0.5649^1^ (37.9, 41.9)	34.53 ± 1.247^1,2^ (30.9, 36.6)	39.38 ± 0.8835^2^ (37.7, 41.0)	F_2, 11_ 12.62 *p* = 0.001^*^
Birth weight (g)	3631 ± 197.9^1^ (2724, 4052)	1989 ± 365.7^1^ (895, 2342)	2944 ± 315.6 (2327, 3632)	F_2, 11_ 8.957 *p* = 0.004^*^
Fetal sex	4 F 6 M	0 F 4 M	4 F 0 M	Chi‐square, *p* = 0.04^*^

*Note*: Matched numbers indicate a significant difference between the two groups. Data show mean ± SEM (min, max).

Abbreviations: F, female; M, male.

*p < 0.05.

**FIGURE 2:**
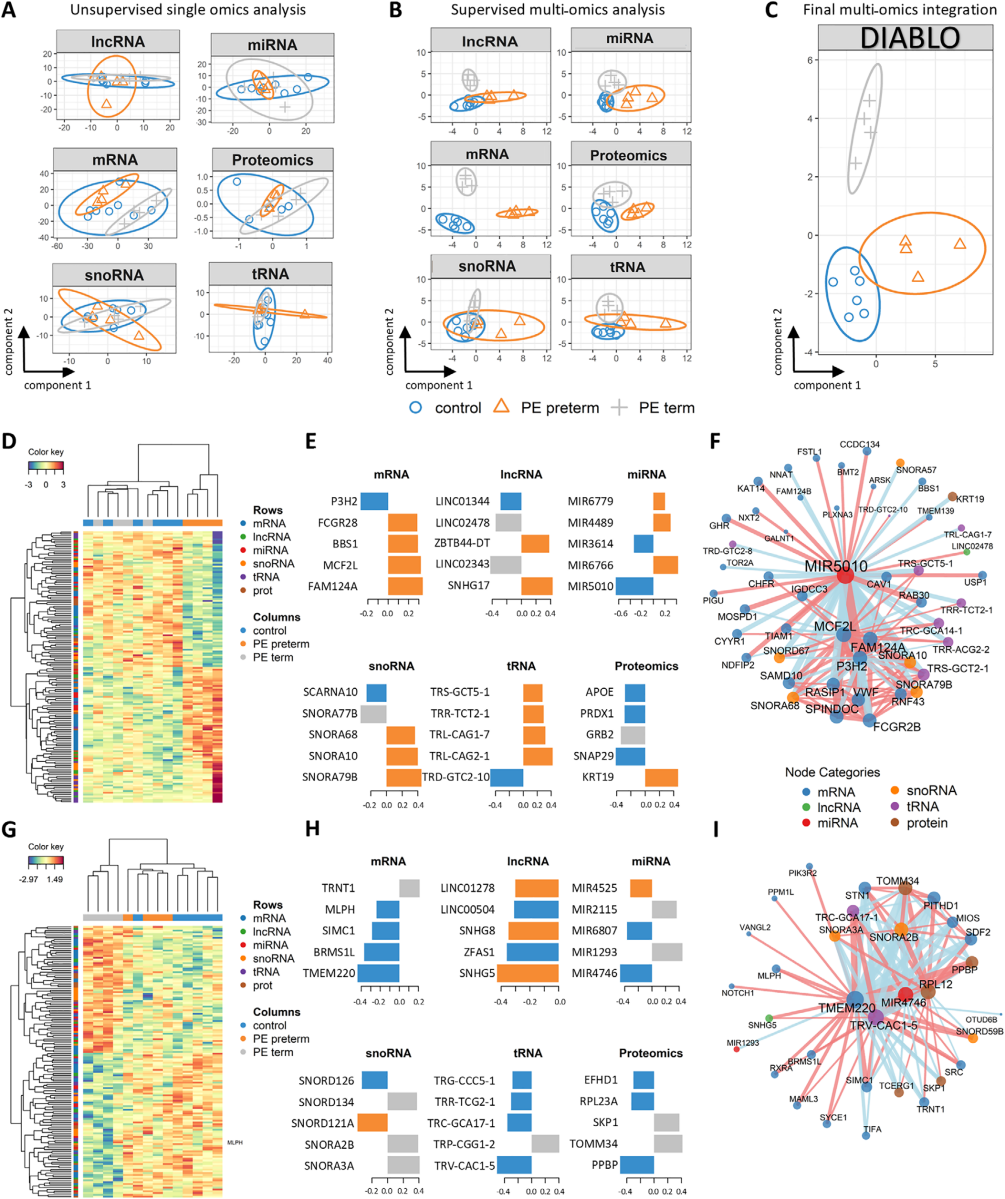
DIABLO identified components that discriminate between preterm preeclampsia, term preeclampsia, and normotensive control CVS. (A–C). Integration of six different omics using the DIABLO method to discriminate between preterm preeclampsia, term preeclampsia, and normotensive control pregnancy groups. X‐axis: component 1 (preterm preeclampsia); Y‐axis: component 2 (term preeclampsia). (A) Preliminary unsupervised analysis with PCA showed indistinct sample clusters according to groups for six different omics (mRNA, lncRNA, miRNA, snoRNA, tRNA, and proteomics). (B) Supervised multi‐omics analysis representing each omics individual shows a clear discrimination between sample groups for most omics except snoRNA. (C) Final multi‐omics integration with DIABLO shows samples cluster according to groups. (D) Heatmap of variables selected by DIABLO that discriminate preterm preeclampsia from term preeclampsia and normotensive controls (first component, X‐axis in C). Variables from different omics are clustered based on their expression similarity across samples. (E) Top 5 selected variables for each omics, with bar length indicating their importance in each omics. Colors indicate the group with the highest average expression level. Full selected variable list can be found in Table S1. (F) Similarity network visualization of the variables selected for preterm preeclampsia by DIABLO. Edge cutoff 0.75 was applied. (G) Similar to (D), but for the second DIABLO component (Y‐axis in C) that discriminates term preeclampsia from preterm preeclampsia and normotensive controls. (H) Similar to (E) for the second DIABLO component. (I) Similar to (F) for the second DIABLO component. Figure created in R.

Integration of six molecular layers (mRNA, lncRNA, miRNA, snoRNA, tRNA, and proteomics) using DIABLO [15] effectively distinguished samples across the three clinical groups (Figure 2A–C). The first component derived from DIABLO (X‐axis in Figure 2C) identified omics variables that primarily discriminated preterm preeclampsia from the other groups (Figure 2D–F; Table S1), while the second component (Y‐axis in Figure 2C) revealed distinct molecular signatures differentiating term preeclampsia samples (Figure 2G–I). These selected variables clustered across omics layers and clearly separated preterm or term preeclampsia cases from the other groups (Figure 2C,D,G). Molecules with the largest absolute loading values in the latent components from DIABLO space are the most influential in driving different subtypes (Figure 2E,H). For preterm preeclampsia, mRNA FAM124A, lncRNA small nucleolar RNA host gene 17 (SNHG17), miRNA MIR5010, snoRNA small nucleolar RNA H/ACA box 79B (SNORA79B), tRNA TRD‐GTC2‐10, and protein cytokeratin 19 (KRT19) were found to be the most influential molecules (Figure 2E). For term preeclampsia, mRNA transmembrane protein 220 (TMEM220), lncRNA SNHG5, miRNA MIR4746, snoRNA SNORA3A, tRNA TRV‐CAC1‐5, and protein pro‐platelet basic protein (PPBP) were the most influential molecules (Figure 2H). The most highly connected molecules identified from the similarity networks are microRNA MIR5010, which was associated with preterm preeclampsia (Figure 2F), and the protein‐coding RNA TMEM220, which was associated with term preeclampsia (Figure 2I). These findings suggest that MIR5010 and TMEM220 may play central regulatory roles in the molecular mechanisms underlying preterm and term preeclampsia, respectively.

The consistency of the DIABLO model was confirmed using leave‐one‐out cross‐validation (LOOCV) (yielding an overall classification error rate of 35.7%). Notably, several key features identified in the original DIABLO model demonstrated high stability across the LOOCV iterations (Figure 2E,H; Figure S1 and Table S1), indicating their consistent importance in distinguishing between the subgroups despite the small sample size.

A sensitivity analysis adjusting for clinical variables confirmed that the sample clustering and the key multi‐omic signatures identified by DIABLO were robust and not significantly influenced by potential confounders (Figure S1). This suggests the multi‐omics model is robust and its findings are not merely driven by these potential confounders.

### 2.2 Molecular Signature of Preterm Preeclampsia

**FIGURE 3:**
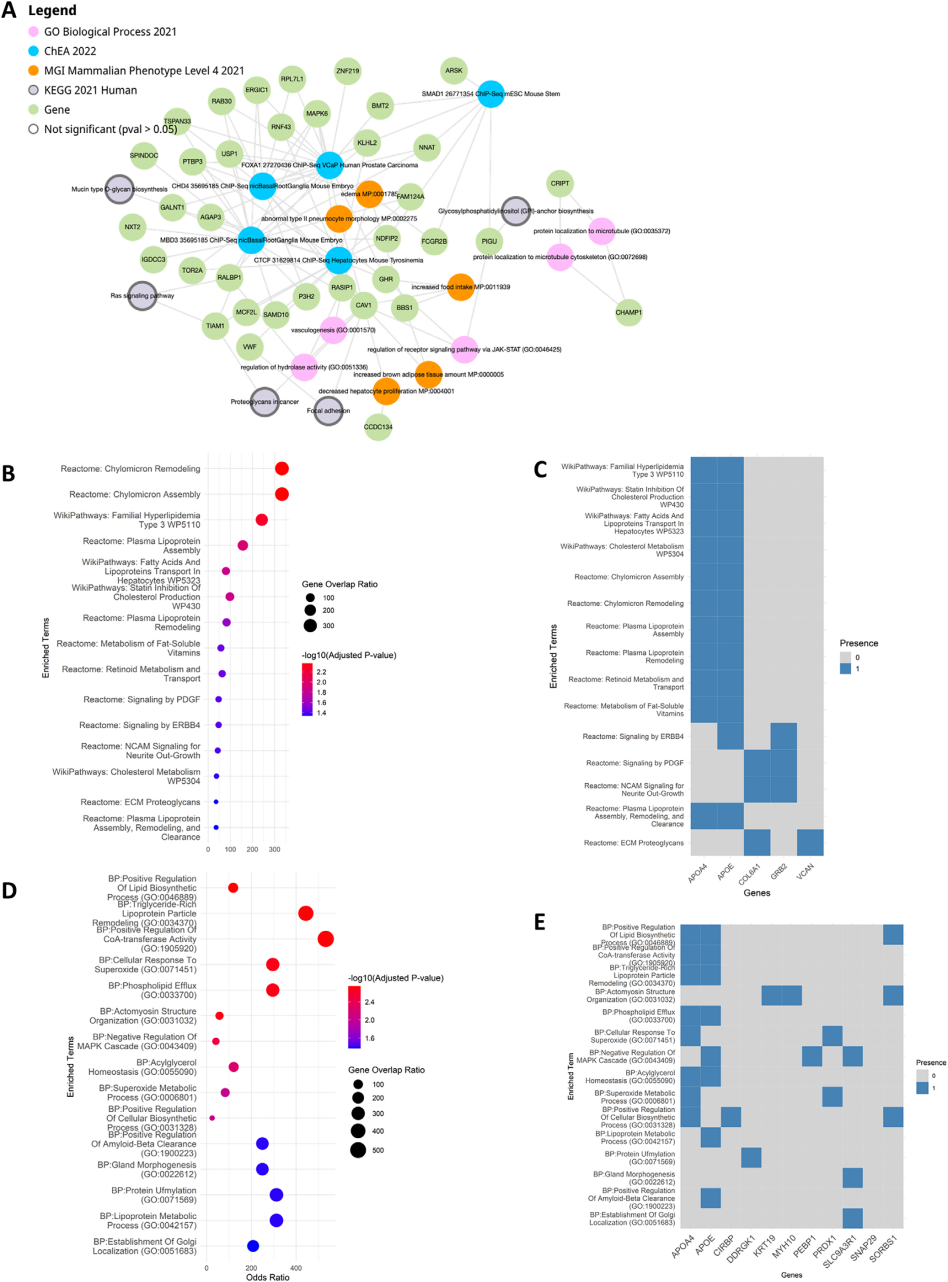
Pathways associated with lipoprotein metabolism were enriched in preterm preeclampsia. (A) Enrichment map connected preterm preeclampsia‐related mRNAs selected by DIABLO with enriched terms from databases. (B) Enriched pathways identified using Wikipathways database from 17 preterm preeclampsia‐related proteins selected by DIABLO. Lipoprotein metabolism pathways (e.g., chylomicron remodeling/assembly, plasma lipoprotein assembly) are significantly enriched. (C) Intersected proteins and enriched terms from Wikipathways were mainly contributed by five well‐studied proteins. (D) Top 20 Gene Ontology (GO) Biological Process (BP) terms that were significantly enriched related to lipoprotein metabolism. (E) Intersected proteins and enriched terms from GO–BP were mainly contributed by 11 proteins. Figure created in R.

Using Enrichr‐KG [16] we created a gene set enrichment map (Figure 3A) to reveal connections between preterm preeclampsia‐associated mRNAs and enriched terms associated with preeclampsia from multiple databases. When we searched the Wikipathways database, no enriched pathways or terms were identified in the 45 mRNAs selected by DIABLO as highly connected with preterm preeclampsia (Table S1). However, many individual genes known to be associated with preeclampsia were identified by DIABLO, including Von Willebrand factor (VWF), caveolin 1 (CAV1), Ras interacting protein 1 (RASIP1), and mitogen‐activated protein kinase 6 (MAPK6) (Table S1).

In contrast, the 17 proteins identified by DIABLO as highly associated with preterm preeclampsia (Table S1) showed significant pathway enrichment (Wikipathways), particularly in lipoprotein assembly and metabolism, as well as platelet‐derived growth factor (PDGF) and erb‐b2 receptor tyrosine kinase 4 (ERBB4) signaling (Figure 3B). This enrichment was driven primarily by five well‐characterized proteins, including apolipoproteins A4 and E (APOA4, APOE), collagen type VI alpha 1 chain (COL6A1), and growth factor receptor‐bound protein 2 (GRB2) (Figure 3C). Gene Ontology (GO) analysis also identified enrichment for lipoprotein metabolism, primarily driven by the proteins APOA4 and APOE (Figure 3D,E; Figure S2).

### 2.3 Molecular Signature of Term Preeclampsia

**FIGURE 4:**
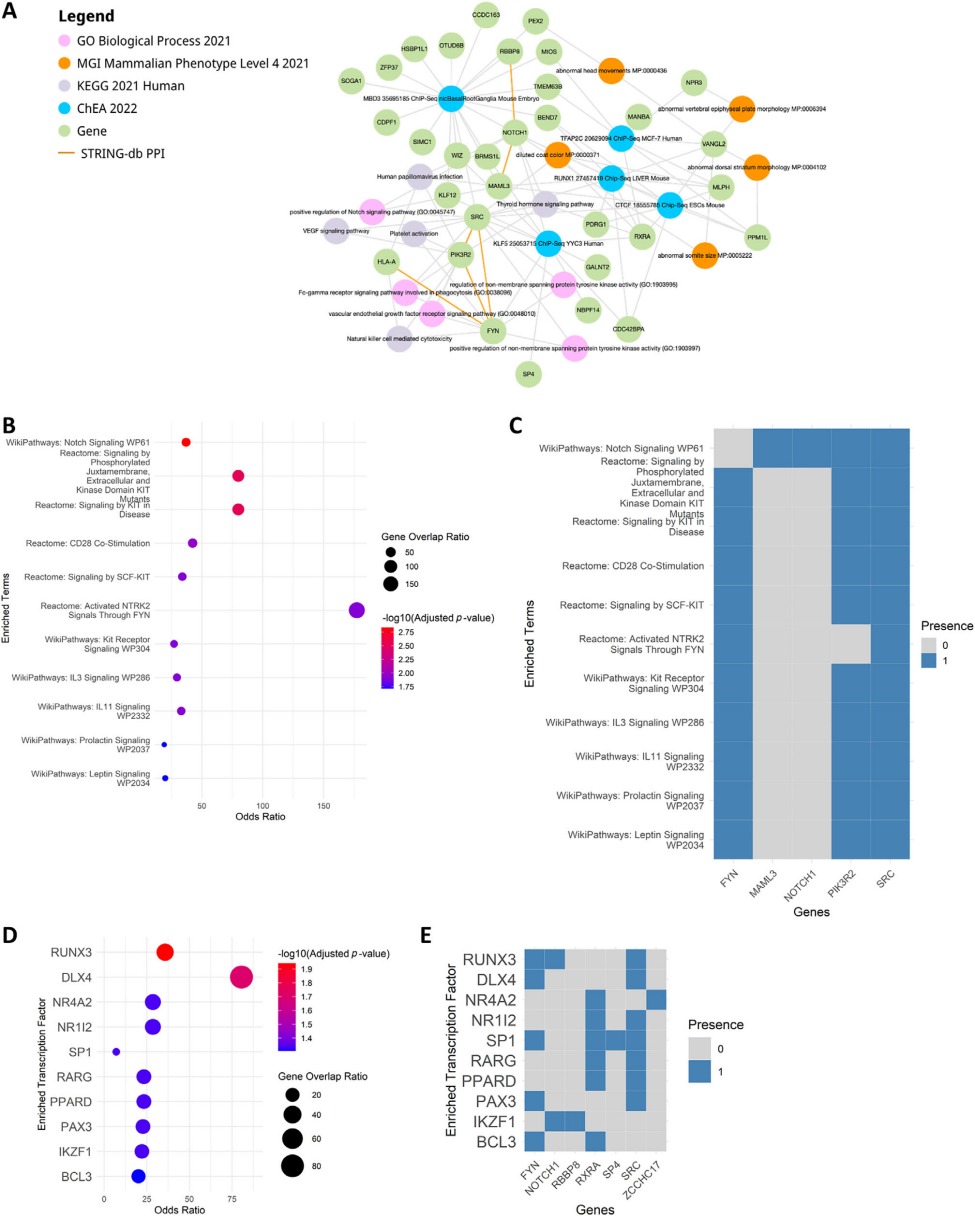
Pathways associated with Notch and inflammatory signaling were enriched in term preeclampsia. (A) Enrichment map connected term preeclampsia‐related mRNA selected by DIABLO with enriched terms from databases. (B) Enriched pathways identified using Wikipathways database from term preeclampsia‐related mRNAs selected by DIABLO. Notch, Kit receptor signaling, inflammatory pathways (e.g., IL‐3, IL‐11), and hormone‐related pathways (e.g., prolactin, leptin) were significantly enriched. (C) Intersected genes and enriched terms from Wikipathways were mainly contributed by five well‐studied genes. (D) Enriched transcription factors identified via overrepresentation analysis of the term preeclampsia‐related mRNA selected by DIABLO and TF‐target gene sets. (E) Four of the genes identified in (D) and a further three genes identified as term preeclampsia‐related share regulatory transcription factors. Figure created in R.

To identify connections between term preeclampsia‐associated mRNAs and enriched terms associated with preeclampsia, we similarly used Enrichr‐KG (Figure 4A) and Wikipathways (Figure 4B–E; Figures S3 and S4). The 45 mRNAs selected by DIABLO as highly connected with term preeclampsia (Table S1) were significantly enriched in signaling cascades such as Notch and Kit receptor signaling, inflammatory pathways like interleukin (IL)‐3 and IL‐11, and hormone‐related pathways such as prolactin and leptin signaling (Figure 4B), all of which have previously been associated with preeclampsia.

The intersected genes and enriched pathways were primarily driven by five well‐characterized genes: FYN proto‐oncogene (FYN), mastermind‐like transcriptional coactivator 3 (MAML3), Notch receptor 1 (NOTCH1), phosphoinositide‐3‐kinase regulatory subunit 2 (PIK3R2), and SRC proto‐oncogene (SRC) (Figure 4C; Figure S3).

Among the 45 genes highly associated with term preeclampsia, numerous transcription factors were identified (Figure 4D), and we found a notable pattern of genes that shared transcriptional regulators (Figure 4E), demonstrating the interconnectedness of the identified factors. Several factors, including MLPH, retinoid X receptor α (RXRA), VANGL planar cell polarity protein 2 (VANGL2), protein phosphatase, Mg^2+^/Mn^2+^‐dependent 1L (PPM1L), and SRC, are all targets of three transcription factors: transcription factor AP‐2 gamma (TFAP2C), RUNX family transcription factor 1 (RUNX1), and CCCTC‐binding factor (CTCF). Further illustrating this connectivity, MLPH, RXRA, and SRC are all linked to the phenotype “diluted coat color (MP: 0000371).”

DIABLO analysis also identified 16 proteins associated with the term preeclampsia (Table S1). Pathway analyses of these proteins revealed enrichment in immune‐related pathways (Figure S4A), primarily mediated by 12 proteins (Figure S4B). The biological processes identified also emphasized the potential involvement of ribosomes (Figure S4C,D).

### 2.4 Validation and Functional Role of MLPH in Term Preeclampsia

To validate the novel factors associated with term preeclampsia discovered in this multi‐omics screen, we interrogated the expression and function of the MLPH, which was previously uncharacterized in the placenta. MLPH was highly associated with term preeclampsia (Figure 2G–I; Table S1). Recent studies have identified MLPH as an adipogenic factor that prevents lipid peroxidation and reactive oxygen species (ROS) accumulation [17]. Based on these findings and our multi‐omics results, we hypothesized that loss of MLPH adversely affects placental villus function, potentially contributing to the pathogenesis of preeclampsia.

**FIGURE 5:**
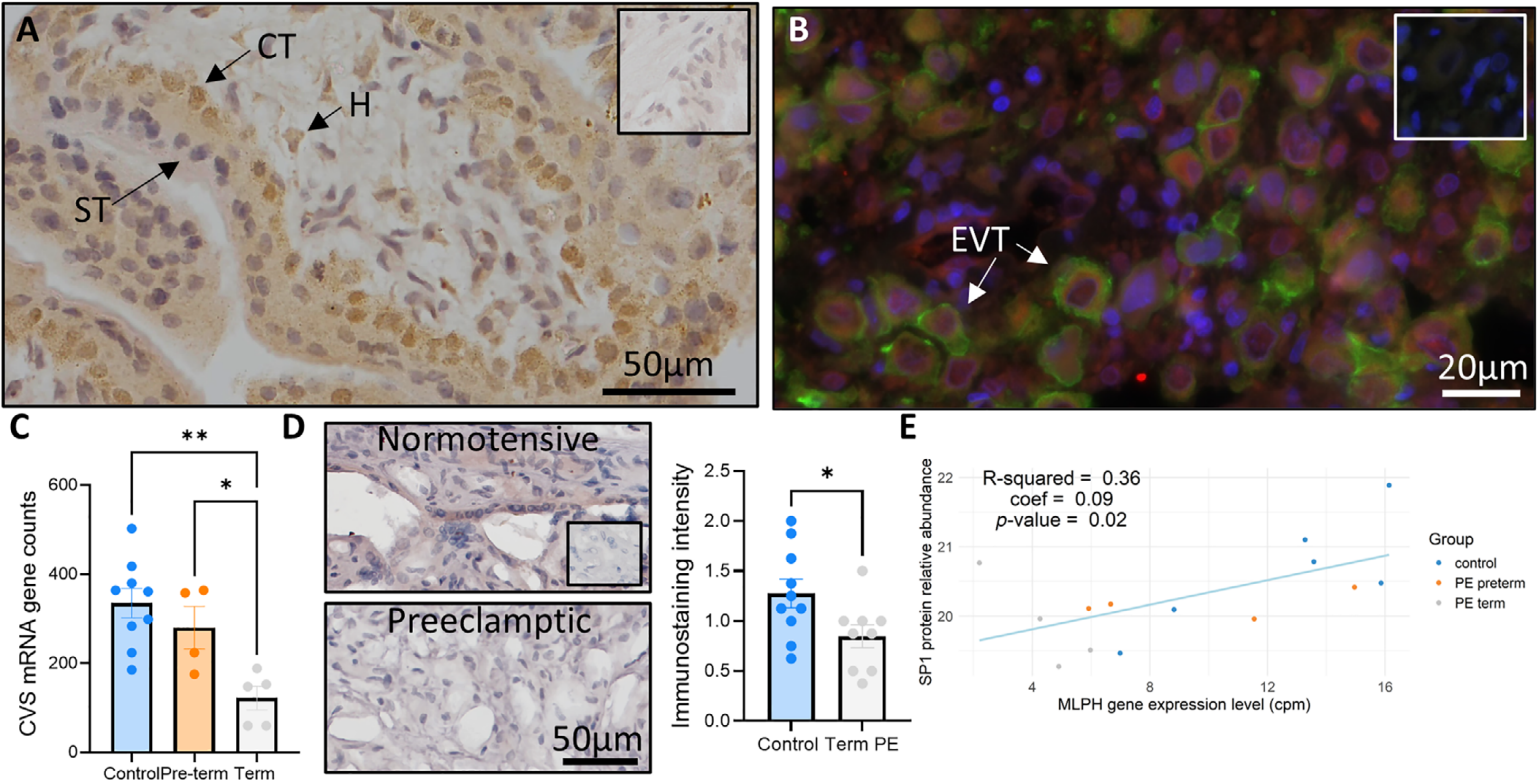
Placental melanophilin expression was reduced in term preeclampsia. (A) MLPH immunostaining in placental villus. MLPH localized to most cells within the placental villus, with strong staining seen in the cytotrophoblast (CT), syncytiotrophoblast (ST), and Hofbauer cells (H). (B) MLPH immunostaining in decidua. MLPH (red) localized to extravillous trophoblast (EVT) identified by HLAG (green) staining. (C) *MLPH* mRNA counts in CVS. (D) MLPH immunostaining was significantly lower in the placenta at delivery from term preeclamptic pregnancies compared to normotensive controls. (E) *MLPH* expression was highly correlated with the transcription factor specificity protein 1 (SP1). Data show mean ± SEM; ^*^ *p* < 0.05; ^**^ *p* < 0.01; statistical tests: (C), one‐way ANOVA; (D), *t*‐test; (E), Pearson correlation. Figure elements created with GraphPad Prism (graphs C,D) and R (E).

MLPH immunolocalization studies in early pregnancy placental villous tissue revealed strong nuclear staining in cytotrophoblasts, while moderate cytoplasmic expression was detected in both cytotrophoblasts and syncytiotrophoblasts (Figure 5A). In addition, cells within the villous core, particularly what appear to be Hofbauer cells, showed notable MLPH expression (Figure 5A). MLPH was produced by most cells in the decidua, including HLA‐G‐positive invasive extravillous trophoblasts (EVTs) (Figure 5B).

Quantitative analysis of *MLPH* mRNA counts (RNA sequencing data) revealed significantly lower expression in CVS from women who developed term preeclampsia compared to both healthy normotensive pregnancies and those who developed preterm preeclampsia (Figure 5C). This differential expression pattern persisted in placentas collected at delivery, with significantly reduced MLPH immunostaining in term preeclamptic placenta tissues compared to normotensive controls (Figure 5D). To explore the potential of MLPH as a biomarker for term preeclampsia risk, we assayed maternal serum MLPH levels across gestation using ELISA; however, MLPH was not readily detectable in maternal serum. We also observed a significant positive correlation between *MLPH* mRNA expression and the protein levels of SP1 transcription factor (SP1) (Figure 5E). SP1 was also strongly associated with term preeclampsia (Figure 4D,E), suggesting a potential regulatory interaction as occurs in melanosomes [18]. The regulatory interaction between SP1 and MLPH may be cell‐type specific, with SP1 negatively regulating *MLPH* expression in melanosomes [18], in contrast to the positive correlation seen here in CVS (Figure 5E). Notably, SP1, like MLPH, is downregulated in preeclampsia [19] and has been identified as an important transcription factor of genes dysregulated in late‐onset preeclampsia, including *11βHSD2*, *CYP19A1*, and *CGB3* [20].

Given the strong expression of MLPH in trophoblasts (Figure 5A,B), we investigated its role in placental function using a human cytotrophoblast progenitor cell (hCTP) line [21] and EVT using HTR8Sv/neo [22] cells.

In hCTP cytotrophoblasts, loss of MLPH (Figure S5A) had no effect on cell proliferation (Figure S5B), cell cycle progression (Figure S5C), or gene expression (Figure S5D,E), except for reduced placental growth factor (PGF) production (Figure S5D).

**FIGURE 6:**
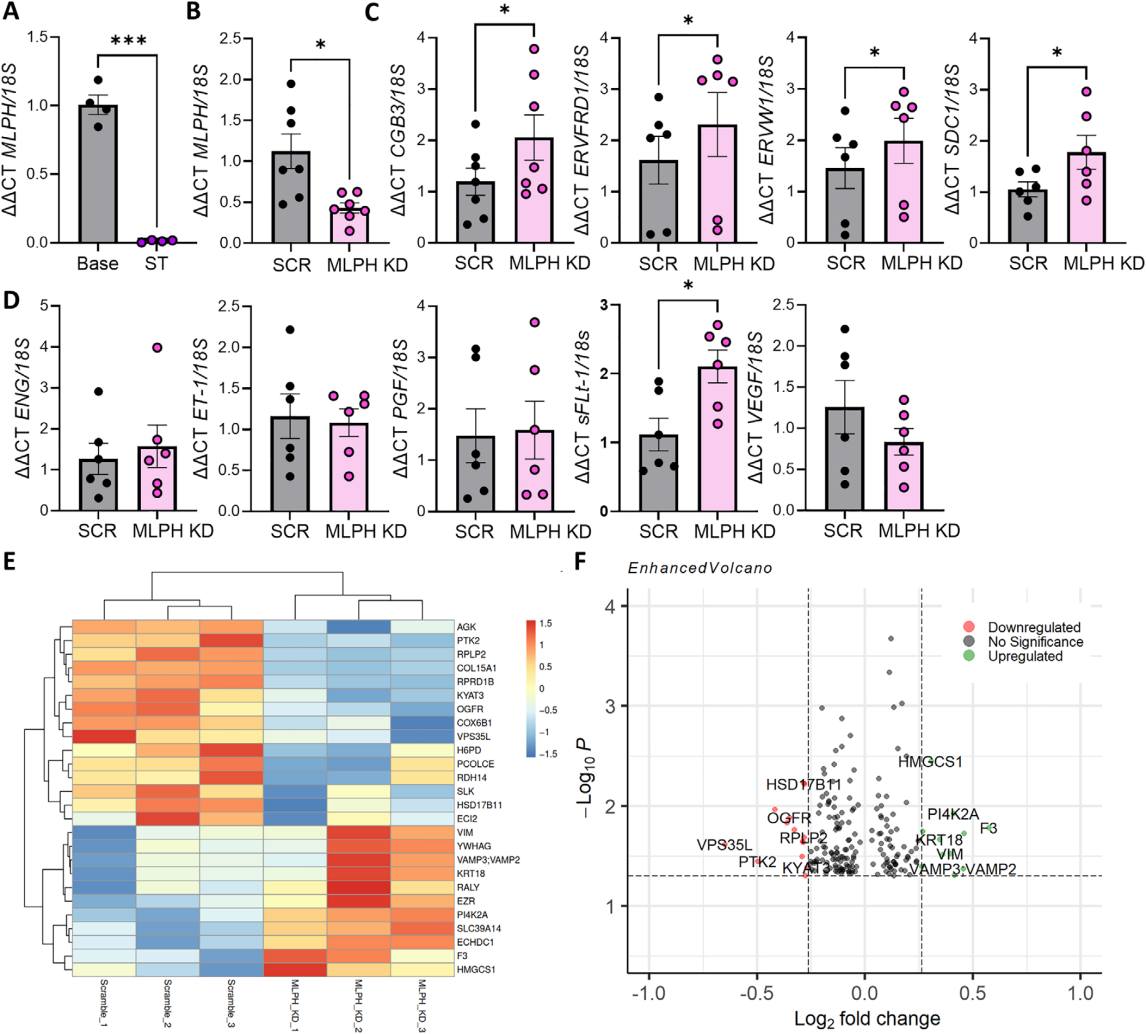
MLPH was required for appropriate syncytial function. (A) *MLPH* production was reduced following syncytialization (ST) of human cytotrophoblast progenitor cells (hCTP) compared to hCTP in base media. (B) *MLPH* production in syncytializing hTCP was further reduced by MLPH knockdown (KD; SCR, scramble siRNA, MLPH, MLPH siRNA). (C) Lower *MLPH* during syncytialization was associated with increased mRNA expression of the syncytialization marker genes *CGB3, ERVFRD, ERVW1*, and *SDC1*. (D) Loss of *MLPH* significantly increased mRNA expression of *sFLT1* but had no effect on other preeclampsia‐associated factors. (E) Heatmap of differentially expressed proteins following syncytialization in control and siMLPH siRNA‐treated hCTP. (F) Volcano plot of differentially expressed proteins following syncytialization in control and knockdown‐treated hCTP. Data show mean ± SEM; ^*^ *p* < 0.05; ^***^ *p* < 0.001; statistical tests: (A–D), paired *t*‐test. Figure created with GraphPad Prism (graphs A–D) and R (E,F).

In vitro syncytialization of hCTP significantly reduced *MLPH* production compared to hCTP cultured in base media (Figure 6A). Reduced *MLPH* production by syncytiotrophoblast was also observed in placental villi, with minimal MLPH immunostaining seen in syncytiotrophoblast compared to cytotrophoblast (Figure 5A). Silencing *MLPH* during hCTP syncytialization (Figure 6B) significantly increased production of the syncytialization‐associated genes chorionic gonadotrophin β (*CGB3*), syndecan 1 (*SDC1*), Syncytin 1 (*ERVW1*), and Syncytin 2 (*ERVFRD1*) (Figure 6C), suggesting a reduction in MLPH is required for syncytialization. However, further investigation revealed that silencing *MLPH* during hCTP syncytialization was also associated with upregulation of the preeclampsia‐associated gene soluble fms‐like tyrosine kinase 1 (*sFLT1*; Figure 6D) but had little effect on other preeclampsia, oxidative stress, or inflammation‐related genes (Figure 6D; Figure S6).

To further explore the impact of excessive loss of *MLPH* during syncytialization, we performed proteomics on hTCP syncytialized following MLPH knockdown. This proteomic analysis revealed significant alterations in the production of 26 proteins (Figure 6E,F; Table S2), many of which have been previously associated with preeclampsia. Notable examples include coagulation factor III (F3), Ezrin, 3‐hydroxy‐3‐methylglutaryl‐CoA synthase 1 (HMGCS1), and protein tyrosine kinase 2 (PTK2).

In the EVT‐like cell line HTR8/SVneo, loss of *MLPH* (Figure S7A) significantly increased adhesion and proliferation (Figure S7B,C) and decreased invasive capacity (Figure S7D), suggesting that loss of *MLPH* was associated with a return to a more cytotrophoblast‐like phenotype.

## 3 Discussion

Our integrated statistical analysis of multiple omics datasets from CVS has provided unprecedented insight into the early pregnancy molecular mechanisms driving preterm and term preeclampsia. This world‐first approach has revealed distinct molecular signatures: preterm preeclampsia was associated with disturbed early pregnancy placental lipoprotein metabolism, whereas term preeclampsia was linked to altered Notch and Kit signaling, inflammatory pathways (including IL‐3 and IL‐11), and ribosome assembly. These molecular changes were identified in placental samples collected months before the onset of maternal preeclampsia symptoms, suggesting a causal rather than consequential role in the disease process.

Term preeclampsia accounts for > 70% of all preeclampsia [1] cases and represents a significant health care burden. Improving its prediction and treatment could substantially impact overall health outcomes and healthcare costs associated with preeclampsia [23]. Our multi‐omics analysis revealed the likely importance of Notch, Kit, and IL‐3 and IL‐11 signaling, as well as ribosome assembly in the etiology of term preeclampsia. These pathways have previously been identified as associated with preeclampsia [24, 25, 26, 27, 28], but this is the first study to identify many of these as dysregulated in early pregnancy.

To validate our multi‐omics findings and provide evidence for their causal role in preeclampsia, we focused on MLPH, a novel highly connected factor identified in term preeclampsia. MLPH production was significantly reduced in both the early pregnancy placental villus (CVS) of pregnancies that subsequently developed term preeclampsia and in placentas collected following term preeclampsia diagnosis (collected at delivery) compared to normotensive controls.

Primarily known for its role in melanocytes, MLPH regulates protein and lipid sorting for melanosome formation and transport through interactions with Rab27a and myosin‐VA [29, 30, 31, 32, 33]. Recent studies have implicated MLPH in vesicle trafficking in other cell types, including insulin granule exocytosis [34], and identified it as an adipogenic factor that prevents lipid peroxidation and ROS accumulation [17]. Therefore, we explored the impact of lowered MLPH on placental function. Our in vitro experiments exploring the function of MLPH in various trophoblast subtypes (cytotrophoblast, syncytiotrophoblast, and EVT) demonstrate that MLPH was critical for normal syncytialization and EVT invasion: both processes, which are disrupted in preeclampsia [1]. Moreover, the increased proliferation coupled with decreased invasion observed in the EVT cell line suggests a potential disruption in the balance between trophoblast self‐renewal and differentiation, which is crucial for proper placental development. This is an entirely new role identified for MLPH.

Following *MLPH* knockdown, syncytialization markers like *CGB3*, *SDC1*, and syncytin 1 and 2 were increased, as were molecules involved in cytoskeletal remodeling and membrane dynamics, including ezrin, vimentin, and keratin 18. This suggests that lowered MLPH facilitates cell fusion. Ezrin, in particular, facilitates the chorionic gonadotrophin β‐stimulated gap junction communication that triggers cytotrophoblast fusion [35]. Overall, our data support a role for MLPH in membrane and vesicle fusion, extending its known roles in insulin granule exocytosis [34] and melanosome transport [30, 31, 32, 33] to include trophoblast syncytialization. These findings align with single‐nucleus multi‐omics profiling of human first‐ and third‐trimester placentas, showing *MLPH* downregulation in the mature syncytiotrophoblast lineage [36].

However, loss of *MLPH* also dysregulated production of many factors known to be disturbed in preeclampsia, including *sFLT1*, coagulation factor III (*F3*) [37, 38, 39], *HMGCS1* [40], cytochrome c oxidase subunit 6B1 (*COX6B1*) [41], and *PTK2* [42] suggesting that while cell fusion is enhanced by lower *MLPH*, excessive loss may lead to syncytial dysfunction, potentially contributing to preeclampsia pathogenesis. F3 was the most increased protein following *MLPH* knockdown in syncytiotrophoblast. F3 is elevated in the plasma of preeclamptic women [43] where it is thought to contribute to a hypercoagulable, anti‐angiogenic and inflammatory state, increasing the production of pro‐inflammatory signals and antiangiogenic factors including sFLT1 [44, 45, 46].

Future research building on these findings has the potential to significantly improve maternal‐fetal health outcomes in term preeclampsia by enabling earlier detection and more effective management of this critical pregnancy complication. Loss of *MLPH* decreased *PGF* and increased *sFLT1* production in cytotrophoblast and syncytiotrophoblast, respectively. An elevated sFLT1/PGF ratio is strongly associated with preeclampsia [47, 48], suggesting that MLPH or its downstream pathways are potential diagnostic or therapeutic targets to identify pregnancies at risk of term preeclampsia or prevent disease.

Our multi‐omics screen also identified pathways associated with the etiology of preterm preeclampsia. Notably, lipoprotein metabolism, including chylomicron remodeling and assembly, was identified as a highly enriched pathway, reflecting the identification of APOA4 and APOE, both previously reported as dysregulated in early‐onset preeclampsia [49, 50].

Our findings may also offer insights into the mechanism by which daily low‐dose aspirin (when initiated before 16 weeks’ gestation) prevents many cases of preterm preeclampsia in high‐risk women [51, 52, 53]. Although the precise mechanism of aspirin's action in preeclampsia prevention remains elusive [54], many factors we found to be highly associated with preterm preeclampsia (including APOE, RALBP1, CAV1, and VWF) are known targets of aspirin in other biological systems [55, 56, 57, 58]. This overlap suggests a potential mechanistic link between aspirin's known effects and its ability to prevent preeclampsia, opening new avenues for targeted therapeutic interventions, ideally ones that are effective even when started after 16 weeks’ gestation.

Attempts to compare our findings here to previous research using CVS are confounded by the samples used and the statistical analyses performed. The original microarray studies published combined samples from both preterm and term preeclampsia [59, 60], and at least one study had decidual contamination [61]. A more recent study used only CVS from preterm preeclampsia (many with fetal growth restriction) to investigate miR expression [62]. No overlapping factors were identified between those three studies or with this study, possibly because of the differences in clinical cohorts. A study using mRNA‐seq to identify differentially expressed genes in CVS from term preeclampsia (*n* = 2) compared to normotensive controls also did not identify any of the factors discovered here [28], although enriched pathways, including IL‐11 signaling and coagulation, were found in both studies. It should also be noted that the aim of the statistical analyses performed in this study was not to identify differentially expressed genes/proteins, but to identify highly correlated factors that differentiate between normotensive pregnancies and preterm and term preeclampsia, making direct comparisons between the specific factors identified in this and previous studies difficult. Encouragingly, however, we did see enrichment of many pathways known to be dysregulated in preeclampsia, supporting our approach.

Factors identified in early pregnancy maternal blood from preeclamptic pregnancies [63, 64] were generally not highly enriched in this study, suggesting that these factors may not come from the placenta or the release of factors by the placenta is biased so only a subset of factors appear differentially produced in circulation.

This study's primary limitation is the small sample size and sex bias of the CVS from preeclamptic pregnancies (Table 1). These constraints are largely due to the increasing rarity of CVS collections worldwide, as noninvasive prenatal testing (NIPT) has decreased the necessity of this procedure. Despite these limitations, our study has uncovered major insights into the early molecular landscape of preeclampsia and represents the only omics study of early pregnancy placental tissue in preeclampsia [59, 65]. Importantly, the factors and enriched pathways we identified align with the current understanding of preeclampsia's molecular mechanisms, lending credibility to our approach. This concordance suggests that our findings, despite the sample size limitations, provide a valuable foundation for future research. Moving forward, validation in larger, more diverse cohorts will be crucial to confirm and extend these results.

In conclusion, our integrated multi‐omics statistical analysis of CVS provides unprecedented insight into the early pregnancy placental molecular landscape, distinguishing preterm and term preeclampsia from normotensive pregnancies. This comprehensive approach has revealed distinct molecular signatures within the early pregnancy placenta from pregnancies that went on to develop preeclampsia: preterm preeclampsia was associated with disturbed lipoprotein metabolism, while term preeclampsia was linked to altered Notch and Kit signaling, inflammatory pathways, and ribosome assembly. Our findings, particularly the validation of MLPH's role in trophoblast function, challenge the belief that term preeclampsia lacks early placental dysfunction. Moreover, the identification of factors associated with preterm preeclampsia that are known targets of aspirin offers potential mechanistic insights into its preventive effects. This study not only advances our understanding of the complex etiology of preeclampsia but also opens new avenues for developing targeted predictive biomarkers and preventative treatments for both preterm and term preeclampsia.

## 4 Materials and Methods

Full methods are provided in Supporting Information.

### 4.1 Placental and Decidual Tissue and Cell Lines Used

Human Research and Ethics Committee approvals (Monash Health and the Royal Women's Hospital, Melbourne #09317B, #95016 and #104524; King's College Hospital, London REC:03‐04‐070; The University of Tokyo #2914‐[4]) were obtained prior to human tissue collection, and written and informed consent was obtained from all participants.

CVS (*n* = 18) were collected between 2010 and 2016 from women undergoing diagnostic testing for fetal chromosomal abnormalities and were immediately snap‐frozen. For our study, we randomly selected samples from chromosomally normal pregnancies that either remained normotensive or subsequently developed preterm or term preeclampsia. These patients were predominantly white (14/18), had spontaneous conception, and were all non‐smokers. Four of 18 were nulliparous, and of the 14 multiparous participants, only one had previously experienced preeclampsia. Patient characteristics for samples included in the multi‐omics analyses (*n* = 14) are shown in Table 1. Preeclampsia was diagnosed by following the American College of Obstetricians and Gynecologists 2019 guidelines [66].

Women undergoing pregnancy termination for psychosocial reasons donated first‐trimester placental villus and decidua tissue (amenorrhea 6–11 weeks; *n* = 4).

Placental samples from women with preeclampsia (and normotensive controls) were collected immediately after delivery from the middle part of the placenta to avoid amnion and decidual contamination (*n* = 9–10/group; Table S3). Preeclampsia was diagnosed by hypertension (blood pressure ≥ 140 mmHg systolic and/or 90 mmHg diastolic after 20 weeks’ gestation) with proteinuria (≤ 300 mg/24 h).

hCTPs were derived from human blastomeres of donated cleavage‐staged embryos [21] and kindly supplied by Susan Fisher (UCSFB‐6; RRID:CVCL_A029). hCTP cells were cultured on 0.5% gelatin (#G1393‐100ml, Sigma) pre‐coated plates/flasks in base media [DMEM/F12 (#10565018, Thermo)] supplemented with 10% FBS, 10 ng/mL basic fibroblast growth factor (#233‐FB‐025, R&D systems, In Vitro Technologies), and 10 µM SB431542 (#1614, Tocris). The EVT cell line (HTR8/SVneo; RRID:CVCL_7162) was purchased from ATCC (#CRL‐3271) and cultured in RPMI 1640 (#11875119, Thermo) supplemented with 10% FBS.

### 4.2 CVS Omics

#### 4.2.1 RNA Sequencing

Full methods are provided in Supporting Information. Briefly, the RNeasy Mini kit (QIAGEN) was used to extract RNA from snap‐frozen tissue and cells before genomic DNA was digested on column (RNase‐free DNase set, #79256, QIAGEN). Spectrophotometry (absorbance ratio: A260/280nm; Nanodrop Thermo Scientific, Scoresby, Victoria, Australia) assessed RNA concentration, yield, and purity. The Australian Genome Research Facility performed the RNA sequencing and primary bioinformatics analysis (Project codes: CAGRF20062989; CAGRF20083494). The RNA sequencing data have been deposited to the National Centre for Biological Information Gene Expression Omnibus (dataset identifier GSE295760).

#### 4.2.2 Proteomics

Full methods are provided in Supporting Information. Briefly, protein was isolated from CVS using TRIZOL as previously described [67] before the protein pellet was washed three times with 0.3 M guanidine hydrochloride (G3272, Sigma) in 95% ethanol, followed by one wash in 100% ethanol, before being prepared for mass spectrometry. For peptide isolation, 3 µg of total cellular protein was used for solid‐phase protein preparation as previously described [68, 69]. An LTQ Orbitrap Elite (Thermo Scientific) coupled to an Ultimate 3000 RSLC nanosystem (Dionex) was used for sample analysis. Samples were assessed according to their label‐free quantification (LFQ) intensities using LFQ‐Analyst [70]. The mass spectrometry proteomics data have been deposited to the ProteomeXchange Consortium PRIDE partner repository (dataset identifier PXD059993) 78

#### 4.2.3 Multi‐Omics Multivariate Analysis

Full methods are provided in Supporting Information. Briefly, we applied the multivariate method DIABLO [15], a supervised, multi‐omics method to identify highly correlated omic variables whose linear combinations (“components”) can discriminate conditions (here control, preterm preeclampsia, and term preeclampsia). Data preprocessing was performed to remove molecules with low expression/missing values [71]. Only samples with matched mRNA, small RNA, and protein data were included in the multi‐omics analysis (*n* = 14). We applied the DIABLO model using two components with a predefined number of selected features for each omics, including 50 mRNAs and 20 for any of the other omics datasets. Our final set of stable and important features included 45 mRNAs, 17 lncRNAs, 19 miRNAs, 17 snoRNAs, 18 tRNAs, 17 proteins associated with preterm, and 47 mRNAs, 13 lncRNAs, 16 miRNAs, 13 snoRNAs, 17 tRNAs, and 17 proteins associated with term preeclampsia (Table S1). The DIABLO analyses generated a similarity matrix for the selected features, which could then be visualized via a bipartite network [72]. Enrichment analysis was performed to identify overrepresented functions or pathways in a set of selected genes [16, 73, 74].

### 4.3 DIABLO Model Validation and Cross‐Validation

To assess the robustness of the DIABLO model, we conducted LOOCV, which is well‐suited for datasets with a small sample size. In this procedure, one sample is excluded from the dataset, and the DIABLO model is trained on the remaining samples with the same parameters described in the above subsection. The sample group of the excluded sample is then predicted using the trained model. This process is repeated 14 times, once for each sample. The overall classification error rate was calculated based on the predictions from all iterations. The stability of the features across the LOOCV was derived as the frequency of each feature being selected in the model across all iterations. The stable features threshold was chosen based on one‐tailed binomial test. Only stable features were reported and included in the downstream analysis.

### 4.4 Sensitivity Analysis for Potential Confounders

To ensure the key findings from the multi‐omics integration analysis were not unduly influenced by the clinical variables and to adjust for the potential selection bias, we performed a sensitivity analysis. We assessed the impact of potential confounders, specifically including gestational age at sample collection time, fetus sex, crown–rump length (CRL), maternal age, BMI, ethnicity (White/other), and parity (nullipara/multipara). Gestational age at delivery and birth weight were not included as they are downstream outcomes of the disease status.

To include the confounders in the DIABLO model, we added the confounders as additional variables to each omics dataset. The DIABLO model was then retrained with the same parameters as described in the “Multi‐Omics Multivariate Analysis” subsection. The sample clustering, feature importance, and selected important feature sets were compared with the original DIABLO model without confounders to evaluate the impact of potential confounders on the multi‐omics integration results.

### 4.5 Melanophilin Validation and Functional Studies

#### 4.5.1 RT‐qPCR

Tissue and cellular RNA were reverse transcribed using Superscript III First‐Strand Synthesis System (Thermo Fisher). qPCR was performed using primers from Sigma‐Aldrich and the Power SYBR Green master mix (Applied Biosystems) on the Viia 7 fast block real‐time qPCR system (Applied Biosystems) in triplicate (final reaction volume, 10 µL) in 384‐well Micro Optical plates (Applied Biosystems). The qPCR protocol was: 95°C for 10 min before 40 cycles of 95°C/15 s, 60°C/1 min. Primer sequences are shown in Table S4. The comparative cycle threshold method (ΔΔCT) was used to calculate relative expression levels.

#### 4.5.2 Immunohistochemistry

Antigen retrieval in boiling Tris‐EDTA buffer for 5 min was followed by peroxidase activity block (3% hydrogen peroxide in methanol, 20 min at RT). Primary antibodies (MLPH Abclonal #A6656, RRID: AB_2767243; first‐trimester villus 4 µg/mL, term villus 5.3 µg/mL) or isotype negative control IgG (Dako X0936) were incubated overnight at 4°C. Sections were washed in Tween‐20 (0.1%) in TBS, before antibody localization was detected by incubation with biotinylated IgG (Goat anti‐Rabbit Vector Laboratories cat# BA1000, RRID: AB_2313606, 1:200 dilution) followed by the avidin–biotin peroxidase complex (Vectastain Elite ABC kit, #PK‐6100), and DAB (Dako K346811‐2) before sections were counterstained with hematoxylin.

#### 4.5.3 Immunofluorescence

Antigen retrieval in boiling 0.01 M sodium citrate buffer for 5 min was followed by primary antibody incubation (MLPH Abclonal #A6656: 2 µg/mL, and HLAG BD BioSciences #557577, RRID: AB_396753: 1 µg/mL) or isotype negative control IgG (Dako X0936, X0931) overnight at 4°C. After stringent washing with Tween‐20 (0.6%) in PBS, antibody localization was detected by the VectaFluor Duet Double Labeling Kit (Vecta, DK‐8828) before sections were counterstained with DAPI (Thermo Fisher Scientific #62248) and mounted (Dako #S3203).

#### 4.5.4 MLPH Silencing

To determine the role of MLPH, hTCP cells cultured in base media were transfected with 80 nM siRNA or scramble control (On‐target plus SMARTPool MLPH: #DHA‐L‐018896‐01‐0005). Knockdown efficiency and the effect on other gene expression were determined by RT‐qPCR at 48 h after transfection.

#### 4.5.5 XTT Assay

At 72, 96, and 120 h after transfection, hTCP cells were treated with XTT reagents and electron coupling reagents (Invitrogen, #X12223) as per the manufacturer's instructions, and cell metabolism was measured by spectrophotometry.

#### 4.5.6 Flow Cytometry

At 48, 72, and 96 h after transfection, hTCP cells were fixed in ice‐cold 70% ethanol, then stained with FxCycle PI/RNase (#F10797, Thermo) and analyzed on a LSR Fortessa flow cytometer (BD BioSciences, USA). The propidium iodide signal was collected using a 610/10 bandpass filter and the yellow/green laser. Doublets were excluded by gating on a YG610/20‐H and YG610/20‐W plot.

#### 4.5.7 hCTP Syncytialization

To induce syncytialization, hCTP cells were cultured on 0.5% gelation in syncytialization media [75] (DMEM/F12 supplemented with 0.5% penicillin–streptomycin, 0.3% BSA, 1% ITS‐X supplement, 2.5 µM Y27632, 2 µM forskolin, and 4% KSR) for 8 days. Cells were photographed before cell pellets, and media were collected and snap‐frozen on Day 8. hTCP syncytialization resulted in morphological changes in the cultured hCTP (Figure S8A) and upregulation of genes associated with syncytialization (Figure S8B; chorionic gonadotrophin β [*CGB3*], syndecan 1 [*SDC1*]) and no change in the expression of genes associated with the cytotrophoblast (Figure S8C) or EVT cell lineage (Figure S8D). To determine the effect of MLPH silencing on hCTP syncytialization, cells were transfected as described above, before the media was replaced with syncytialization media 48 h after transfection. Cells were photographed before cell pellets, and media were collected and snap‐frozen on Day 6 after transfection.

#### 4.5.8 hCTP Proteomics

Briefly, total protein was extracted from cells in RIPA buffer (#89900, Thermo) plus protease inhibitor (#539134, Merck Millipore) and phosphatase inhibitor (#5870S, Cell Signaling Technologies) by mechanical homogenization (QIAGEN TissueLyser; RIPA buffer). A total of 15µg of total cellular protein was utilized for proteomics. The analysis was performed on an Orbitrap Ascend mass spectrometer (Thermo Scientific) coupled with a nano‐flow reversed‐phase HPLC system (Ultimate 3000 RSLC, Dionex). Raw LC‐MS/MS data files were processed and analyzed using MaxQuant‐Andromeda v2.6.2.0. For quantification, LFQ intensities were used, with the “match between runs” option enabled to maximize protein identification across runs. Statistical evaluation of the proteomic data was carried out using Perseus software (2.0.11). Proteins were considered differentially expressed if their LFQ intensity fold change was greater than 1.2 or less than 0.83, with statistical significance set at *p* < 0.05. Proteins with at least two peptides were considered for differential expression analysis.

#### 4.5.9 Adhesion/Proliferation Assay

The real‐time cell analyzer (RTCA) MP xCELLigence instrument (ACEA Biosciences; Agilent Technologies GmbH) was used to assess cell adhesion and growth [76]. Briefly, following 72 h of MLPH or scramble control knockdown, 10,000 HTR8/Svneo cells were seeded (E‐plate 96, ACEA Biosciences; Agilent Technologies GmbH) in media supplemented with 5% FBS. Cell adhesion and growth were monitored every 15 min for the first 8 h, then every hour for a total of 84 h.

#### 4.5.10 Invasion Assay

The RTCA DP xCELLigence instrument (ACEA Biosciences; Agilent Technologies GmbH) was used to assess cell invasion [77]. Briefly, following 72 h of MLPH or scramble control knockdown, 30,000 HTR8/Svneo cells were seeded into the upper chamber of a CIM‐plate 16 pre‐coated with Matrigel (1:10 dilution in RPMI 1640; ACEA Biosciences; Agilent Technologies GmbH) in media supplemented with 5% FBS. Medium supplemented with 15% FBS was added to the lower chamber. Plates were monitored every hour for a total of 48 h.

## Author Contributions

E.M., K.L.C., and E.D. were responsible for conception and design of the study. A.S., K.K., D.L.R., F.S.A., K.L.C., K.N., and E.D. acquired tissues and clinical and surgical data. S.V. and N.W. undertook proteomics assays and performed statistical analyses of the proteomics data. G.Y. and K.L.C. performed statistical and bioinformatics analyses. E.M., Y.Y., L.L.S., and W.Z. performed the experiments. All authors have read and approved the final manuscript.

## Funding

Research reported in this publication was supported in part by the National Health and Medical Research Council (NHMRC Australia): Project Grant GNT2019920 and Investigator Grants GNT2025648 (K.L.C.) and GNT2025670 (D.L.R.); the University of Melbourne Department of Obstetrics, Gynaecology and Newborn Health Early‐Career/Mid‐Career Research Fellowships (E.M., W.Z.), the University of Melbourne Department of Obstetrics, Gynaecology and Newborn Health Innovation Grant; the China Scholarship Council–University of Melbourne PhD Scholarship (G.Y.); the Melbourne Research Scholarship (Y.Y.); the University of Melbourne MDHS Research Grant Support Scheme; The Norman Beischer Medical Research Foundation; the Trevor B Kilvington Bequest; the Rowden White Trust; and The Victorian Government's Operational Infrastructure Support Program. Support for CVS sample collection and storage was provided by the UK charity, The Fetal Medicine Foundation.

## Ethics Statement

Human placental tissue was collected under appropriate Human Research and Ethics Committee approvals (Monash Health and the Royal Women's Hospital, Melbourne #09317B, #95016 and #104524; King's College Hospital, London REC:03‐04‐070; The University of Tokyo #2914‐[4]). Written and informed consent was obtained from all participants.

## Conflicts of Interest

E.M., G.Y., K.L.C., and E.D. have lodged a patent filing for biomarkers of preeclampsia risk. E.M. declares financial support from Bayer outside the submitted work. The other authors declare no conflicts of interest.

## Supporting information




**Supporting File 1**: mco270784‐sup‐0001‐SupMat.docx

**Supporting File 2**: mco270784‐sup‐0002‐TableS1.xlsx

**Supporting File 3**: mco270784‐sup‐0003‐TableS2.xls

## Data Availability

The RNA sequencing data have been deposited in the National Centre for Biological Information Gene Expression Omnibus (dataset identifier: GSE295760). The mass spectrometry proteomics data have been deposited in the ProteomeXchange Consortium PRIDE [78] partner repository (dataset identifier: PXD059993).
