# Supplementary material for: Multi‐Omics Reveals Early Pregnancy Placental Dysfunction Associated With Preterm and Term Preeclampsia
Source: MedComm (2020). 2026 May 26;7(6):e70784. doi: 10.1002/mco2.70784 (PMC13240503; doi:10.1002/mco2.70784)
Supplement: Supplementary file 1 — Supporting File 1: mco270784‐sup‐0001‐SupMat.docx [file MCO2-7-e70784-s003.docx]

**Supplementary data.**

**Multi-omics reveals early pregnancy placental dysfunction associated with preterm and term preeclampsia.**

Short title: Early placental dysfunction of preeclampsia

Ellen Menkhorst^1,2^, Guannan Yang^1,2,3^, Yimiao Yu^1,2^, Leilani L Santos^1,2^, Wei Zhou^1,2^, Argyro Syngelaki^4,5^, Swati Varshney^6^, Nicholas Williamson^6^, Kaori Koga^7,8^, Daniel Lorber Rolnik^9,10^, Fabricio da Silva Costa^11,12^, Kypros Nicolaides^4,5^, Kim-Anh Lê Cao^3^, Evdokia Dimitriadis^1,2*.^

^1^Department of Obstetrics, Gynaecology and Newborn Health, The University of Melbourne, Parkville, VIC, Australia

^2^Gynaecology Research Centre, Royal Women’s Hospital, Parkville, VIC, Australia

^3^Melbourne Integrative Genomics, School of Mathematics and Statistics, The University of Melbourne, Parkville, VIC, Australia

^4^Fetal Medicine Foundation, London, UK

^5^Harris Birthright Research Centre for Fetal Medicine, King’s College Hospital, London, United Kingdom

^6^Melbourne Mass Spectrometry and Proteomics Facility, Bio21 Molecular Science & Biotechnology Institute, The University of Melbourne, Melbourne, VIC, Australia

^7^Department of Obstetrics and Gynecology, The University of Tokyo, Tokyo, Japan

^8^Department of Obstetrics and Gynecology, Reproductive Medicine, Chiba University, Chiba, Japan

^9^Department of Obstetrics and Gynaecology, Monash University, Melbourne, Victoria, Australia

^10^Women’s and Newborn, Monash Health, Melbourne, Victoria, Australia

^11^Maternal Fetal Medicine Unit, Gold Coast University Hospital, Gold Coast, Queensland, Australia

^12^ School of Medicine and Dentistry, Griffith University, Gold Coast, Queensland, Australia

*Corresponding author: Evdokia Dimitriadis, Level 7, Royal Women’s Hospital, 20 Flemington Rd, Parkville, VIC, Australia, 3052; Phone: +61 3 8345 2215; email: evdokia.dimitriadis@unimelb.edu.au

Supplementary Methods

### CVS RNA sequencing: RNA isolation

RNA was extracted from snap-frozen tissue and cells using the RNeasy Mini kit (QIAGEN) according to the manufacturer’s instructions. Genomic DNA was digested on column (RNase-free DNase set, #79256, QIAGEN) according to the manufacturer’s instructions. RNA concentration, yield and purity were analyzed by spectrophotometry (Nanodrop Thermo Scientific, Scoresby, Victoria, Australia) at an absorbance ratio of A260/280nm.

### CVS RNA sequencing: Sequencing

After the RNA libraries (total RNA, small RNA) were prepared using Illumina’s Ribo-zero Gold protocol, they were sequenced on an Illumina NovaSeq platform: 150bp paired-end run (total RNA)/100bp single end run (small RNA). Following primary bioinformatics analysis (demultiplexing and quality control) the data was processed through RNA-seq expression analysis workflow, including alignment, transcript assembly, quantification and normalization. The cleaned sequence reads were then aligned against the *Homo sapiens* genome (Build version HG38) using STAR aligner (v2.5.3a). In total, 39,533 RNA (including mRNA and small RNA) were read and identified. Two samples were not included in further analysis: 1 was removed due to rRNA contamination (1 sample, 15%) and lowest percentage of reads mapping to the genome (73.47%), the other was removed due to only 36.08% of reads mapping to one feature.

### CVS Proteomics: Protein isolation using TRIZOL

Protein was isolated from CVS using TRIZOL as previously described.^1^ The organic phase was mixed with two volumes of isopropyl alcohol (I9516, Sigma), incubated at room temperature for 10 min, and centrifuged at 10,000 g for 10 min.

### **CVS Proteomics: Peptide isolation**

3ug total cellular protein was used for Solid-Phase Protein Preparation as previously described.^2, 3^ SP3 protocol was carried with 10μg of extracted protein samples in a total volume of 50μL Triethyl ammonium bicarbonate buffer (TEAB) followed by reduction with 10 mM TCEP for 45 minutes at 37°C and alkylation with 55mM Iodoacetamide for 45 minutes at 37°C in dark. Magnetic beads were prepared by combining 20μL of both Sera-Mag Speed Beads A and B (GE Healthcare cat. no. 45152105050250; cat. no. 65152105050250) and washed two times with 200μL ddH2O and were re-suspend in 40μL ddH2O for a final working concentration of 50μg/μL. 2μL of pre-washed magnetic beads as well as 50μL 100% ethanol were added to each sample. Protein binding to the beads was facilitated in ThermoMixer at 24°C for 5 min at 1,000 r.p.m. After the binding is complete, tubes were placed in a magnetic rack and were incubated until the beads have migrated to the tube wall. The supernatant was removed, and beads were washed thrice with 180μL of 80% ethanol. Beads were resuspended in 100μL of 100 mM TEAB and sonicated for 5 minutes in a water bath. The samples were then kept for overnight digestion at 37°C and 1000 rpm in a table-top thermomixer after adding sequencing-grade trypsin in an enzyme:protein ratio of 1:10. Upon digestion, peptides were recovered by collecting the supernatant. These peptides were lyophilized and stored until mass spectrometry analysis.

CVS Proteomics: LC-MS/MS

Samples were reconstituted in 30 μL 2% acetonitrile:0.1% trifluoroacetic acid and were analysed on a LTQ Orbitrap Elite (Thermo Scientific) coupled to an Ultimate 3000 RSLC nanosystem (Dionex). The nanoLC system was equipped with an Acclaim Pepmap nano-trap column and an Acclaim Pepmap analytical column. 3 μl of the peptide mix was loaded onto the trap column at 3% CH3CN containing 0.1% formic acid for 5 min before the enrichment column is switched in-line with the analytical column. The LC gradient used was 3% B to 23% B for 89 min, 23% B to 40% B in 10 min, 40% B to 80% B in 5 min and maintained at 80% B for the final 5 min before equilibration for 10 min at 3% B prior to the next analysis. The LTQ Orbitrap Elite mass spectrometer was operated in the data-dependent mode, with spectra acquired first in positive mode at 240k resolution followed by collision induced dissociation (CID) fragmentation. Twenty of the most intense peptide ions with charge states ≥2 were isolated and fragmented using normalized collision energy of 35 and activation Q of 0.25 (CID).

### CVS Proteomics: Analysis

Raw data files were searched against the Human protein reference proteomes (UniProt Proteome ID: [UP000005640](https://www.uniprot.org/proteomes/UP000005640)) using MaxQuant-Andromeda (version 1.6.7.0). The false discovery rate (FDR) was set at 0.01 for both peptides and proteins. Search parameters were set as follows: variable modifications: Oxidation (M), Acetyl (Protein N-term); fixed modifications: cysteine carbamidomethylation. The analysis of the samples was based on the label-free quantification (LFQ) intensities. Initial analyses and visualization of proteomics data was performed using LFQ-Analyst.^4^ The data was statistically evaluated using Perseus software (version 1.6.7.0). The protein data was filtered categorically by row for reverse identifications (false positives), contaminants, and proteins “only identified by site”.

### CVS Multi-omics multivariate analysis: Data preprocessing

Genes were typed based on HGNC database (<https://www.genenames.org/download/statistics-and-files>, retrieved on 24/08/2023). Low expression RNA were filtered out, leaving 12148 mRNAs, 338 lncRNAs, 675 miRNAs, 200 snoRNAs and 274 tRNAs. Trimmed mean of M-values (TMM) and Counts per million (CPM) was used for normalization. Proteins in the proteomic data were filtered by the numbers of missing values. Proteins with more than five missing values in 14 samples were filtered out, leaving 616 proteins. NIPALS (Non-linear Iterative PArtial Least Squares)^5^ was used to impute the missing values with the number of components of 12. Then, the proteomic data were log transformed and normalised by VSN (Variance Stabilizing Normalization).^6^ Only samples with matched mRNA, small RNA and protein data were included in the multi-omics analysis (n=14).

### **CVS Multi-omics multivariate analysis: DIABLO multivariate analysis**

We applied the multivariate method DIABLO (Data Integration Analysis for Biomarker discovery using Latent cOmponents),^6^ a supervised, multi-omics method to identify highly correlated omic variables whose linear combinations (‘components’) can discriminate conditions (here control, preterm preeclampsia and term preeclampsia). We applied DIABLO model using 2 components with a predefined number of selected features for each omics, including 50 mRNAs and 20 for any the other omics datasets.

Association measure between datasets and the response (control, term and pre-term) was set to 0.6 with mRNA and moderate with the other datasets (0.05, 0.10, 0.05, 0.05, 0.15 for lncRNA, miRNA, snoRNA, tRNA, proteomics respectively). Association between pairwise omics datasets was set to 0.05, according to our preliminary analyses of agreements between datasets. Leave-one-out (LOO) cross-validation was used to determine the stability of the selected omic variables. To further strengthen the feature selection results, we established a stability threshold, defined as the minimum frequency with which a feature must be selected to confidently reject the hypothesis that its selection was due to random chance, using a binomial model. Our final set of stable and important features included 45 mRNAs, 17 lncRNAs, 19 miRNAs, 17 snoRNAs, 18 tRNAs, 17 proteins associated with preterm and 47 mRNAs, 13 lncRNAs, 16 miRNAs, 13 snoRNAs, 17 tRNAs and 17 proteins associated with term preeclampsia (Table S1). Heatmaps of the expression or relative abundance levels of these variables were generated. Hierarchical clustering was performed on both variables and samples using complete linkage method with Euclidean distance.

### **CVS Multi-omics multivariate analysis: Similarity Network**

The DIABLO analyses generated a similarity matrix for the selected features, which could then be visualised via a bipartite network.^7^

### CVS Multi-omics multivariate analysis: Enrichment Analysis

Enrichment analysis was performed to identify overrepresented functions or pathways in a set of selected genes. Functional annotations were obtained from Gene Ontology (GO), Wikipathway and Transcription factor protein-protein interaction (TF-PPI), accessed Enrichr web platform^8^ using overrepresentation analysis with a hypergeometric test (FDR < 0.05). GOSemSim^9^ was used to reduce the redundancy in the enrichment result towards GO database based on semantic similarity of the enriched GO terms, which was determined by hierarchical clustering and the most representative terms from clusters (cut at a height of 0.7) were selected. Enrichment network visualisation was obtained using Enrichr-KG.^10^

### hCTP Proteomics: Protein isolation

Total protein was extracted from snap-frozen tissue by mechanical homogenization (QIAGEN Tissue Lyser; RIPA buffer (#89900, Thermo) plus protease inhibitor (#539134, Merck Millipore) and phosphatase inhibitor (#5870S, Cell Signaling Technologies) before cell membranes were pelleted at 10,000x*g* and protein quantified (Pierce BCA assay, ThermoFisher Scientific #23225).

### hCTP Proteomics: Peptide isolation

Fifteen micrograms of total cellular protein were utilized for hCTP proteomics. The proteins were diluted and sonicated for 5 minutes, then centrifuged at 13,000xg for 8 minutes. The resulting cell pellet was resuspended in 10 mM TCEP (pH 7.0, Sigma cat# 646547) and incubated at 37°C for 45 minutes to reduce disulfide bonds. Alkylation of peptides was carried out by adding 55 mM iodoacetamide (Sigma cat# I1149-5G) and incubating in the dark at room temperature for 45 minutes. Phosphoric acid (2.5% final concentration, Sigma #79617) was then added, and the samples were briefly vortexed. After dilution with binding buffer, proteins were captured using S-Trap micro columns (ProtiFi S-Traps micro columns). Digestion was performed with sequencing-grade trypsin at a 1:20 dilution.

### hCTP Proteomics: LC-MS/MS

Samples were reconstituted in 30 μL of 2% acetonitrile/0.1% trifluoroacetic acid. The analysis was performed on an Orbitrap Ascend mass spectrometer (Thermo Scientific) coupled with a nano-flow reversed-phase HPLC system (Ultimate 3000 RSLC, Dionex. The nano-LC system was equipped with an Acclaim PepMap nano-trap column (C18, 100 Å, 75 μm × 2 cm) and an Acclaim PepMap RSLC analytical column (C18, 100 Å, 75 μm × 50 cm). For each run, 1 μL of peptide mixture was loaded onto the trap column at a flow rate of 5 μL/min in an isocratic solution of 2% acetonitrile with 0.05% trifluoroacetic acid for 6 minutes, before switching the flow to the analytical column. The mobile phase consisted of two solvents: Solvent A (0.1% formic acid in water) and Solvent B (100% acetonitrile/0.1% formic acid). A gradient elution was applied as follows: 3% B to 23% B over 89 minutes, 23% B to 40% B over 10 minutes, and 40% B to 80% B over 5 minutes. The gradient was maintained at 80% B for 5 minutes, followed by equilibration at 3% B for 10 minutes before the next sample injection. The mass spectrometer operated in positive ion mode. Full-scan MS spectra were acquired from m/z 375 to 1500 with a resolution of 120K and an automatic gain control (AGC) target of 400,000, with a maximum injection time of 50 ms. Peptide ions with charge states ranging from 2 to 7 were selected for fragmentation using higher-energy collisional dissociation (HCD) with a normalized collision energy of 30. MS2 spectra were acquired at a resolution of 15K, with an AGC target of 50,000 and a maximum injection time of 22 ms. The isolation window was set to 1.6 m/z.

### hCTP Proteomics: Analysis

Raw LC-MS/MS data files were processed and analyzed using MaxQuant-Andromeda v2.6.2.0). The data were searched against the Human protein reference proteome database (UniProt Proteome ID: UP000005640, Swiss-Prot, Reviewed). Search parameters included variable modifications such as oxidation (M) and acetylation (Protein N-term), while cysteine carbamidomethylation was set as a fixed modification. Trypsin were specified as the proteolytic enzymes, allowing for a maximum of two missed cleavages. The false discovery rate (FDR) was set at 0.01 for both peptides and proteins. For quantification, label-free quantification (LFQ) intensities were used, with the "match between runs" option enabled to maximize protein identification across runs. Statistical evaluation of the proteomic data was carried out using Perseus software (2.0.11). Reverse identifications, contaminants, and proteins identified "only by site" were excluded from the analysis. Proteins were considered differentially expressed if their LFQ intensity fold change was greater than 1.2 or less than 0.83, with statistical significance set at p < 0.05. Proteins with at least two peptides were considered for differential expression analysis.

**References**

1. Kopec AM, Rivera PD, Lacagnina MJ, Hanamsagar R, Bilbo SD. Optimized solubilization of TRIzol-precipitated protein permits Western blotting analysis to maximize data available from brain tissue. *J Neurosci Methods*. 2017;280:64-76.

2. Dagley L, Infusini G, Larsen RH, Sandow JJ, Webb AI. Universal Solid-Phase Protein Preparation for Bottom-up and Top-down Proteomics. *Journal of Proteome Research*. 2019;18(7):2915-2924.

3. Hughes C, Moggridge S, Müller T, Sorensen PH, Morin GB, Krijgsveld J. Single-pot, solid-phase-enhanced sample preparation for proteomics experiments. *Nature Protocols*. 2019;14(1):68-85.

4. Shah AD, Goode RJA, Huang C, Powell DR, Schittenhelm RB. LFQ-Analyst: An Easy-To-Use Interactive Web Platform To Analyze and Visualize Label-Free Proteomics Data Preprocessed with MaxQuant. *Journal of Proteome Research*. 2020;19(1):204-211.

5. Martens H, Martens M. *Multivariate analysis of quality: an introduction*. John Wiley & Sons; 2001.

6. Singh A, Shannon CP, Gautier B, et al. DIABLO: an integrative approach for identifying key molecular drivers from multi-omics assays. *Bioinformatics*. 2019;35(17):3055.

7. Gonzalez I, Le Cao K-A, Davis MD, Dejean S. Insightful graphical outputs to explore relationships between two 'omics' datasets. *BioData Mining*. 2013;5:19.

8. Chen EY, Tan CM, Kou Y, et al. Enrichr: interactive and collaborative HTML5 gene list enrichment analysis tool. *BMC bioinformatics*. 2013;14:128.

9. Yu G, Li F, Qin Y, Bo X, Wu Y, Wang S. GOSemSim: an R package for measuring semantic similarity among GO terms and gene products. *Bioinformatics*. 2010;26(7):976-8.

10. Evangelista JE, Xie Z, Marino GB, Nguyen N, Clarke DJB, Ma'ayan A. Enrichr-KG: bridging enrichment analysis across multiple libraries. *Nucleic Acids Res*. 2023;51(W1):W168-w179.

Table S1. Highly correlated molecules identified by multi-omics (DIABLO) that discriminate between preterm preeclampsia, term preeclampsia and control groups.

Excel file uploaded separately.

Table S2. Proteins dysregulated in syncytialized hCTP following MLPH knockdown.

Excel file uploaded separately.

Table S3. Maternal characteristics of placentas used for immunohistochemistry.

|  | Control (n=10) | Preeclampsia (n=9) |  |
| --- | --- | --- | --- |
| **Maternal age** | 31.80+1.22 (25, 37) | 31.11+1.84 (27, 39) | t=0.404, df 17, p=0.69 |
| **Gestational age at delivery** | 38.76+0.36 (37, 41) | 37.90+0.77 (35, 41) | t=1.032, df 17, p=0.32 |
| **Parity** | 0.45+0.16 (0, 1) | 0.13+0.13 (0, 1) | t=1.541, df 17, p=0.14 |
| **Mode of delivery** | 3 CS, 7 V | 7 CS, 2 V | Chi Sq, p=0.07 |
| **Staining intensity by delivery method** | CS 1.42+0.29; V 1.21+0.17 | CS 0.82+0.15; V 1.8+0.94 |  |

Data shows mean+sem (min, max); CS, C-section; V, vaginal.

Table S4. Primer sequences.

| **Gene** | **Forward primer 5`-3`** | **Reverse primer 5`-3`** |
| --- | --- | --- |
| *18S* | GATCCATTGGAGGGCAAGTCT | CCAAGATCCACCTACGAGCTT |
| *CDX2* | GACGTGAGCATGTACCCTAGC | GCGTAGCCATTCCAGTCCT |
| *CGB3* | ATGGACTCGAAGCGCACATC | GCTACTGCCCCACCATGACC |
| *CYBA* | ACCGGGTTTATGATATTCCACCT | GATTTCGACAGACTGGCAAGA |
| *CYBB* | ACCGGGTTTATGATATTCCACCT | GATTTCGACAGACTGGCAAGA |
| *ENG1* | TTTGTCTTCGGCAGTGCTTACT | TTTTCCGCTGTGGTGATGA |
| *ERVFRD1* | AGCCTTAACGACCATGCAAGA | CTGTGCTGCCGTTAACATGTC |
| *ERVW1* | CCCCATCGTATAGGAGTCTT | CCCCATCAGACATACCAGTT |
| *ET-1* | GTCAACACTCCCGAGCACGTT | CTGGTTTGTCTTAGGTGTTCCTC |
| *FLT1* | CGTAGAGATGTACAGTGAAA | GGTGTGCTTATTTGGACATC |
| *sFLT1* | CTCCTGCGAAACCTCAGTG | GACGATGGTGACGTTGATGT |
| *GEMININ* | GCCCTGGGGTTATTGTCCC | AGCGCCTTTCTCCGTTTTTCT |
| *HLAG* | GAGGAGACACGGAACACCAAG | GTCGCAGCCAATCATCCACT |
| *ICAM1* | ATGCCCAGACATCTGTGTCC | GGGGTCTCTATGCCCAACAA |
| *IL6* | TTTCAGCCATCTTTGGAAGG | TACCCCCAGGAGAAGATTCC |
| *IL18* | GCATCAACTTTGTGGCAATG | TCCGGGGTGCATTATCTCTA |
| *ITGA6* | TGCTGTTGGTTCCCTCTCAGAT | CTGGCGGAGGTCAATTCTGT |
| *MLPH* | AGGGAGACTCAGATGACTCCA | CCCCAGTATCAGCCTCCTC |
| *NCF1* | GGGGCGATCAATCCAGAGAAC | GTACTCGGTAAGTGTGCCCTG |
| *NCF2* | CCCACTCCCGGATTTGCTTC | GTCTCGGTTAATGCTTCTGGTAA |
| *NOS3* | TGATGGCGAAGCGAGTGAAG | ACTCATCCATACACAGGACCC |
| *PAPPA2* | CCTCACCTAGAGACTCCTTGG | AGGGGATAGTCCTATTGGGCA |
| *PGF* | CCCTTGGGTCTCCTCCTTTC | TGCTGCGGCGATGAGAATC |
| *RAC1* | ATGTCCGTGCAAAGTGGTATC | CTCGGATCGCTTCGTCAAACA |
| *SDC1* | CTGCCGCAAATTGTGGCTAC | TGAGCCGGAGAAGTTGTCAGA |
| *VCAM1* | GGGAAGATGGTCGTGATCCTT | TCTGGGGTGGTCTCGATTTTA |
| *VEGF* | AGGGCAGAATCATCACGAAGT | AGGGTCTCGATTGGATGGCA |

**
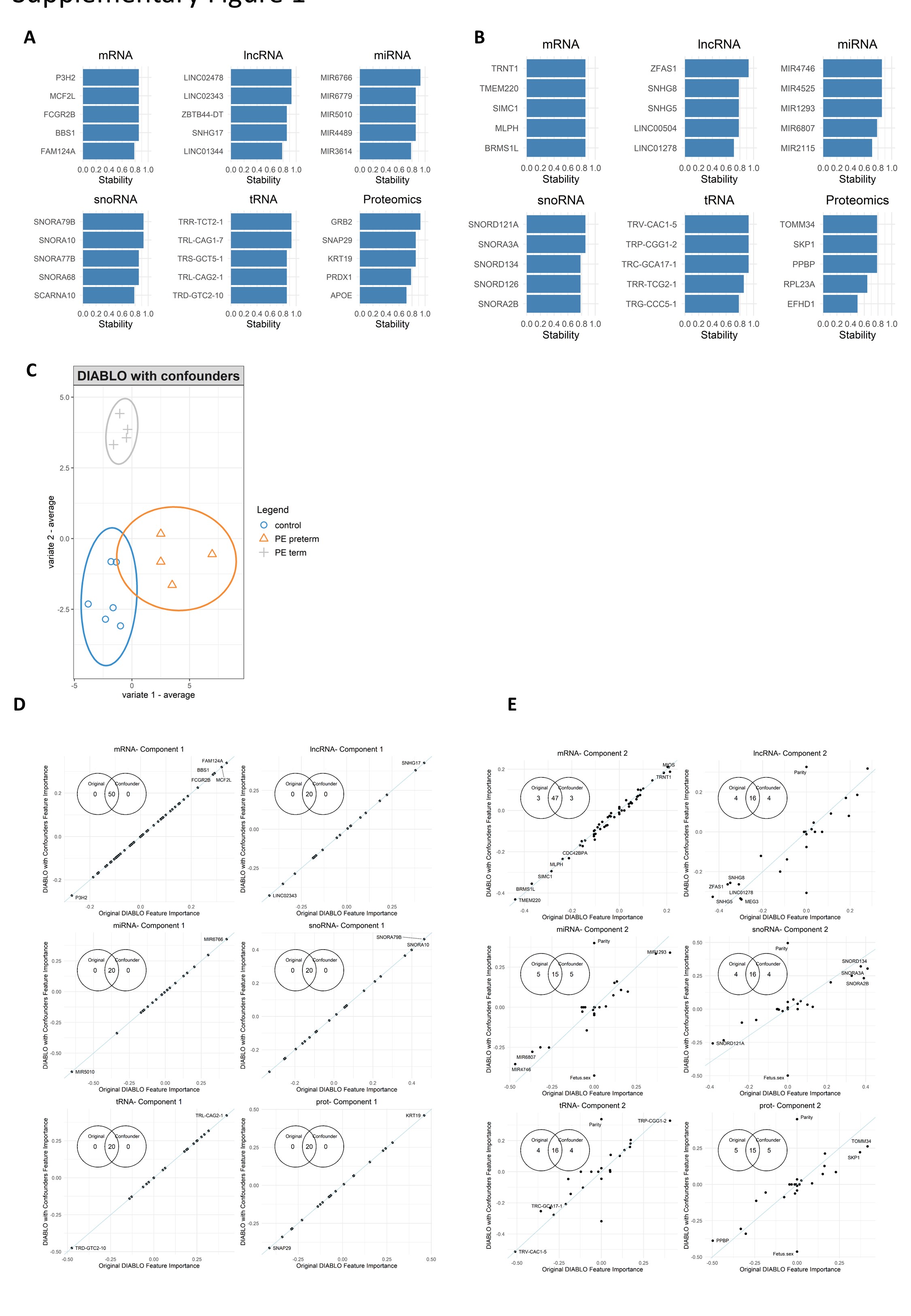
**

**Figure S1.**

Figure S1. **Evaluation the consistency of DIABLO model results.** A-B. Feature stability across cross-validation iterations. A. Stability of top 5 variables with the highest importance in component 1 across the leave-one-out cross-validation (LOOCV). The length of the bar indicates the frequency of the feature being selected in the DIABLO model across all LOOCV iterations. Full selected variable list can be found in Supplementary Table 1. B. Stability of top 5 variables with the highest importance in component 2 across the LOOCV. C-E. Results of a sensitivity analysis on the DIABLO model with potential confounders, including gestational age at sample collection time, fetus sex, crown-rump length (CRL), maternal age, BMI, ethnicity (White/other) and parity (nullipara/multipara). C. Sensitivity analysis adjusting for potential confounders showing similar sample clustering as the original DIABLO model (Figure 2C). Each point represents a sample, colored by the disease status. The axes represent the first two components derived from the DIABLO model incorporating confounders. The subgroups remain significantly seperated by the first two components ($p < 0.001$, PERMNOVA). D. Scatter plots showing high correlation of feature importance (selected in component 1) between the original DIABLO model and the DIABLO model adjusted for potential confounders. Each point represents a feature. The axes represent feature importance in the original model (x-axis) and the confounder-adjusted model (y-axis). The blue line (y = x) indicates the equality of the importances from two models. The venn plots show the overlap of important features (importance $>$ 0) selected in component 1 between the two models for each omics. The high correlations and overlapping suggest that the importance of features in component 1 (majorly discriminates preterm preeclampsia), is largely unaffected by the inclusion of potential confounders. E. Scatter plots showing the correlation of feature importance (selected in component 2) between the original DIABLO model and the DIABLO model adjusted for potential confounders. Figure elements are the same as in Supplementary Figure 4D. Variables with importance equal to zero indicate that these features were not selected in the respective DIABLO model. The moderate correlations suggest that the importance of features in component 2 (majorly discriminates term preeclampsia), is somewhat influenced by the inclusion of potential confounders. It is notable that the clinical variables `fetus sex' and `parity' were among the top important features in component 2 in all omics, which might be caused by the unbalance distributions of these two variables in the term preeclampsia and the other groups. Correspondingly, the inclusion of these variables in the DIABLO model affected the importance of the omic features. Most of the omic features still showed similar importance in both models, with only a few features' importance changing considerably. Figure created using R.


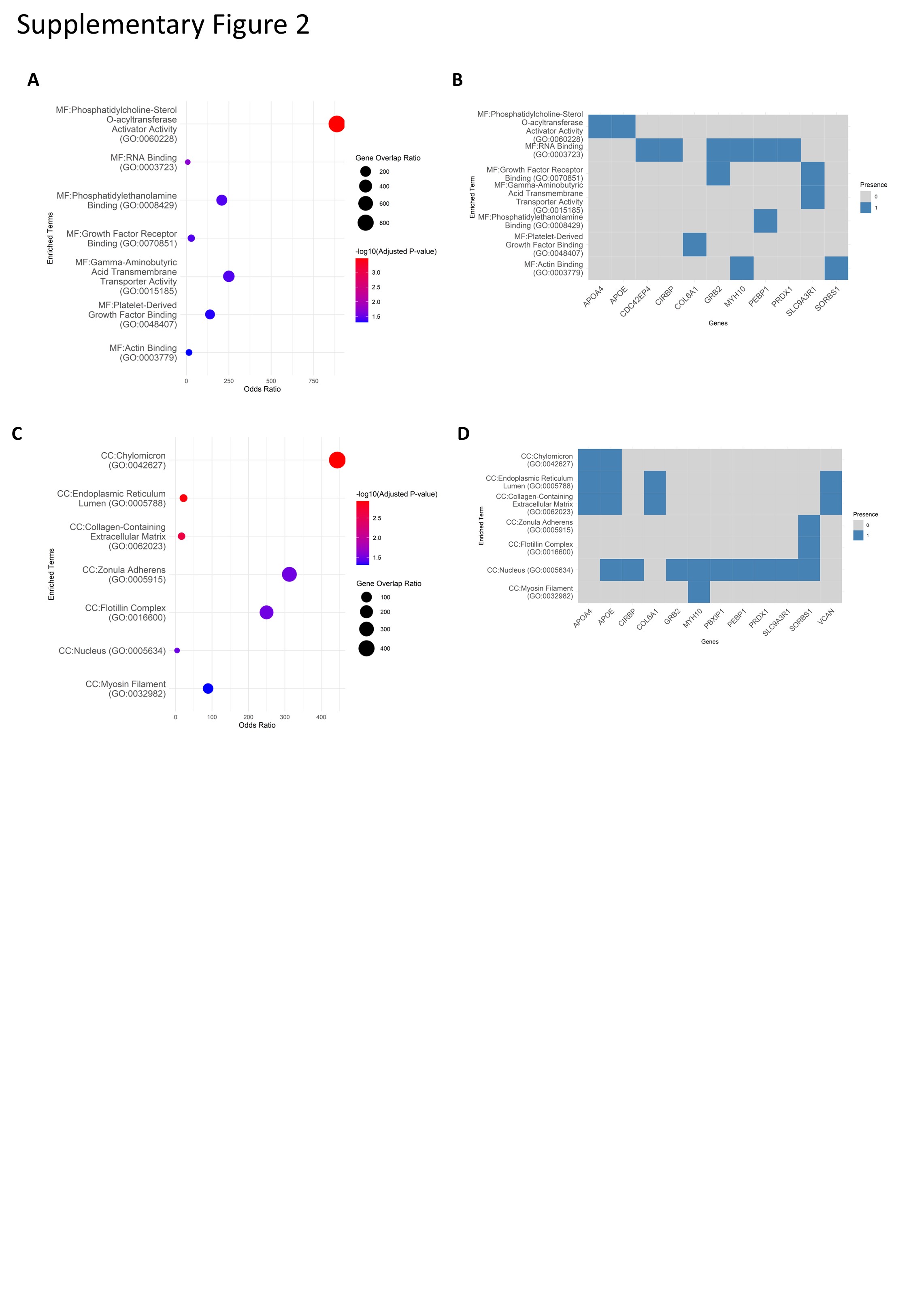


Figure S2. Enriched Gene Ontology terms in preterm preeclampsia-related proteins. A. Gene Ontology (GO) Molecular Function (MF) terms which were significantly enriched in preterm preeclampsia. B. Intersected proteins and enriched terms from GO-CC were mainly contributed by 11 proteins. C. Gene Ontology (GO) Cellular Components (CC) terms which were significantly enriched in preterm preeclampsia. D. Intersected proteins and enriched terms from GO-CC were mainly contributed by 12 proteins. Figure created using R.


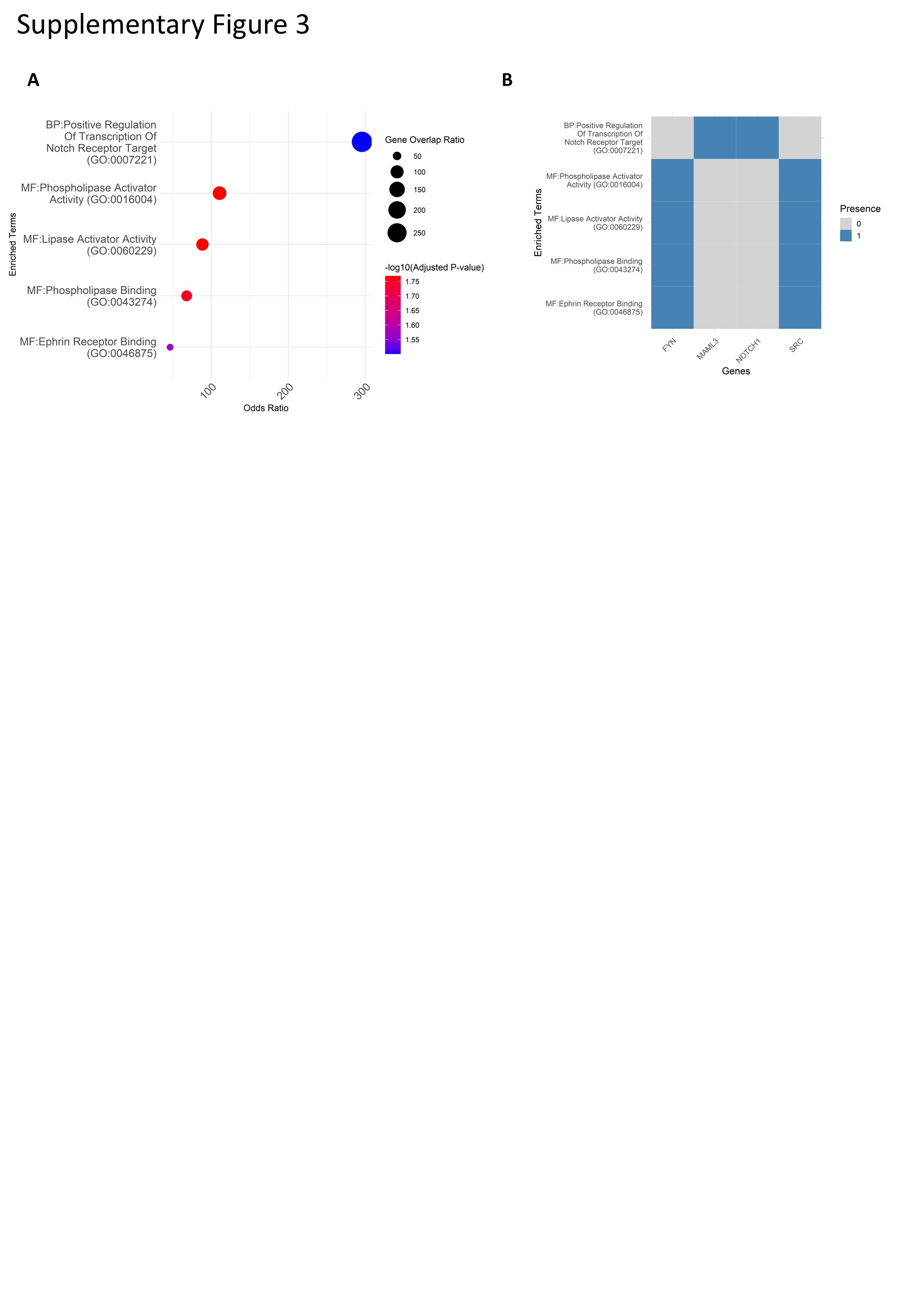


Figure S3. Enriched Gene Ontology terms in term preeclampsia-related mRNA. A. Gene Ontology (GO) terms which were significantly enriched in term preeclampsia. B. Intersected genes and enriched terms from GO were mainly contributed by 4 proteins. Figure created using R.


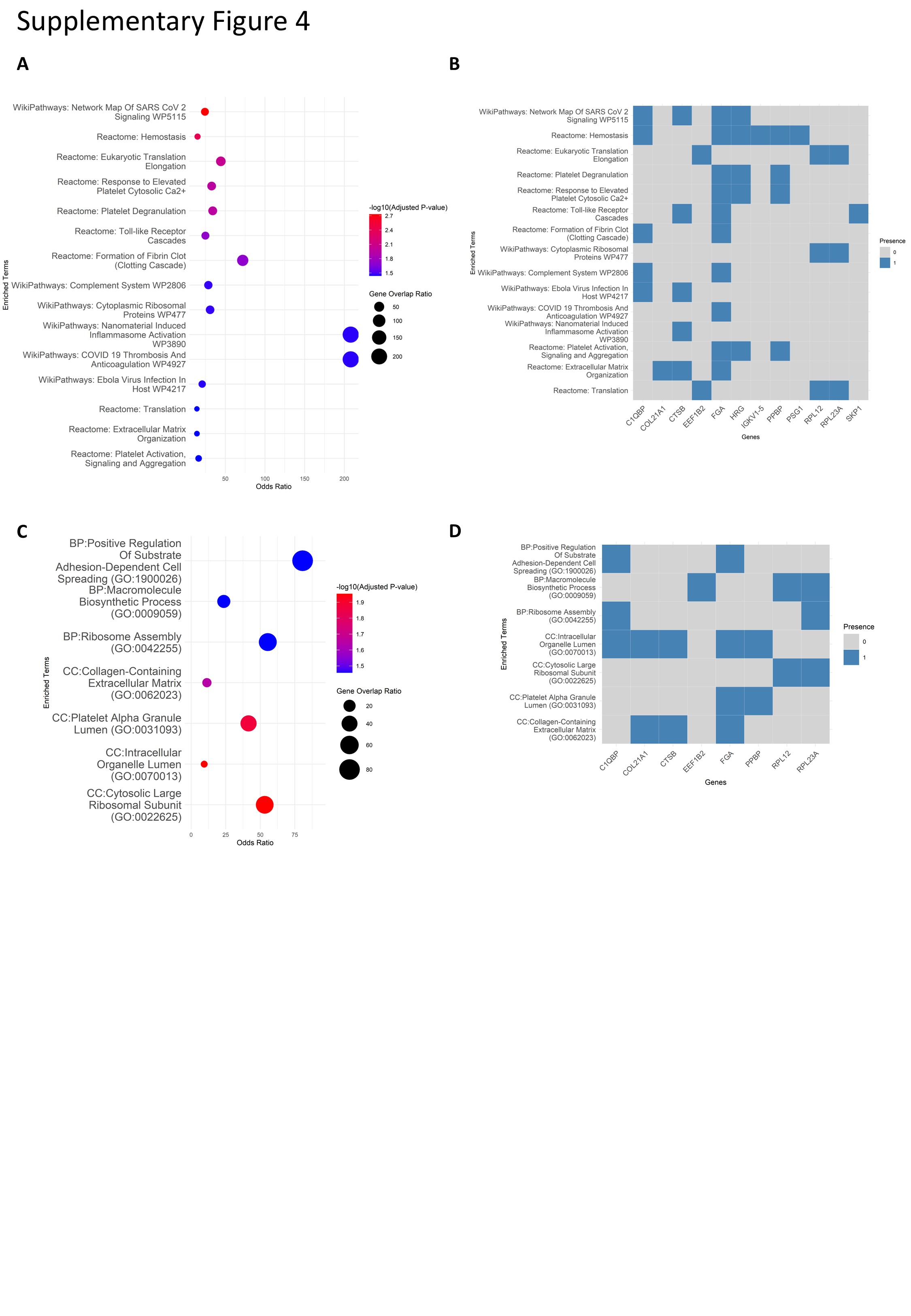


Figure S4. Enriched pathways and Gene Ontology terms in term preeclampsia-related proteins. A. Pathways enriched by term-preeclampsia-related proteins selected from component 2. B. Intersected proteins and enriched pathways were mainly contributed by 12 proteins. C. Gene Ontology (GO) terms which were significantly enriched. D. Intersected proteins and enriched terms from GO were mainly contributed by 8 proteins. Figure created using R.


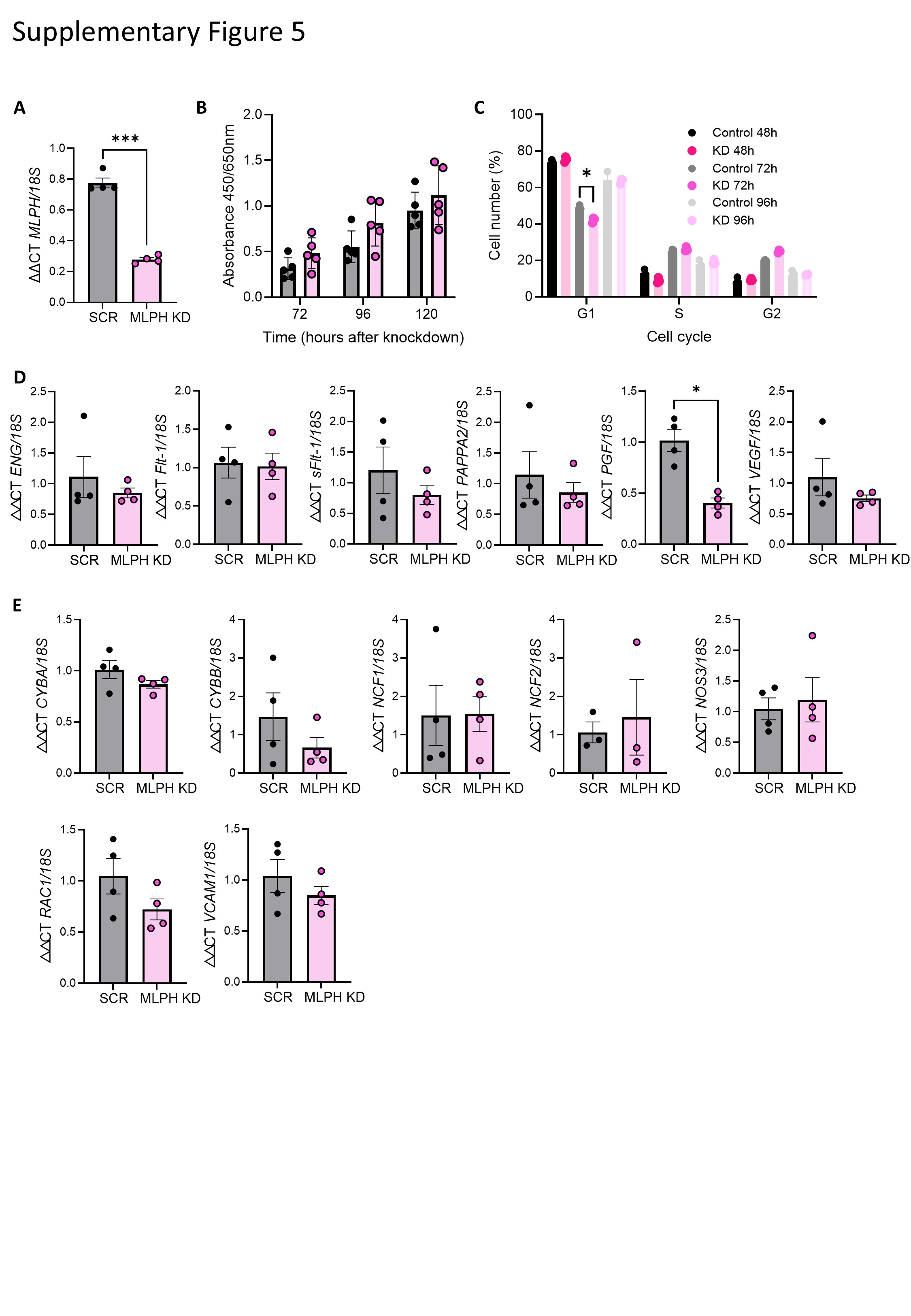


Figure S5. MLPH had little effect on cytotrophoblast progenitor cell (hTCP) function. A. MLPH knockdown (KD; SCR, scramble siRNA, MLPH, MLPH siRNA)in hTCP had no effect on B. cell proliferation by XTT assay or C. cell cycle progression. D-E. Loss of MLPH significantly reduced D. PGF production but had no other effect on D. preeclampsia-related or E. oxidative-stress related gene expression. Data shows mean+sem; *, p<0.05; ***, p<0.001; Statistical tests: A,D-E, paired t-test; B&C, two-way anova. Figure created using GraphPad Prism.


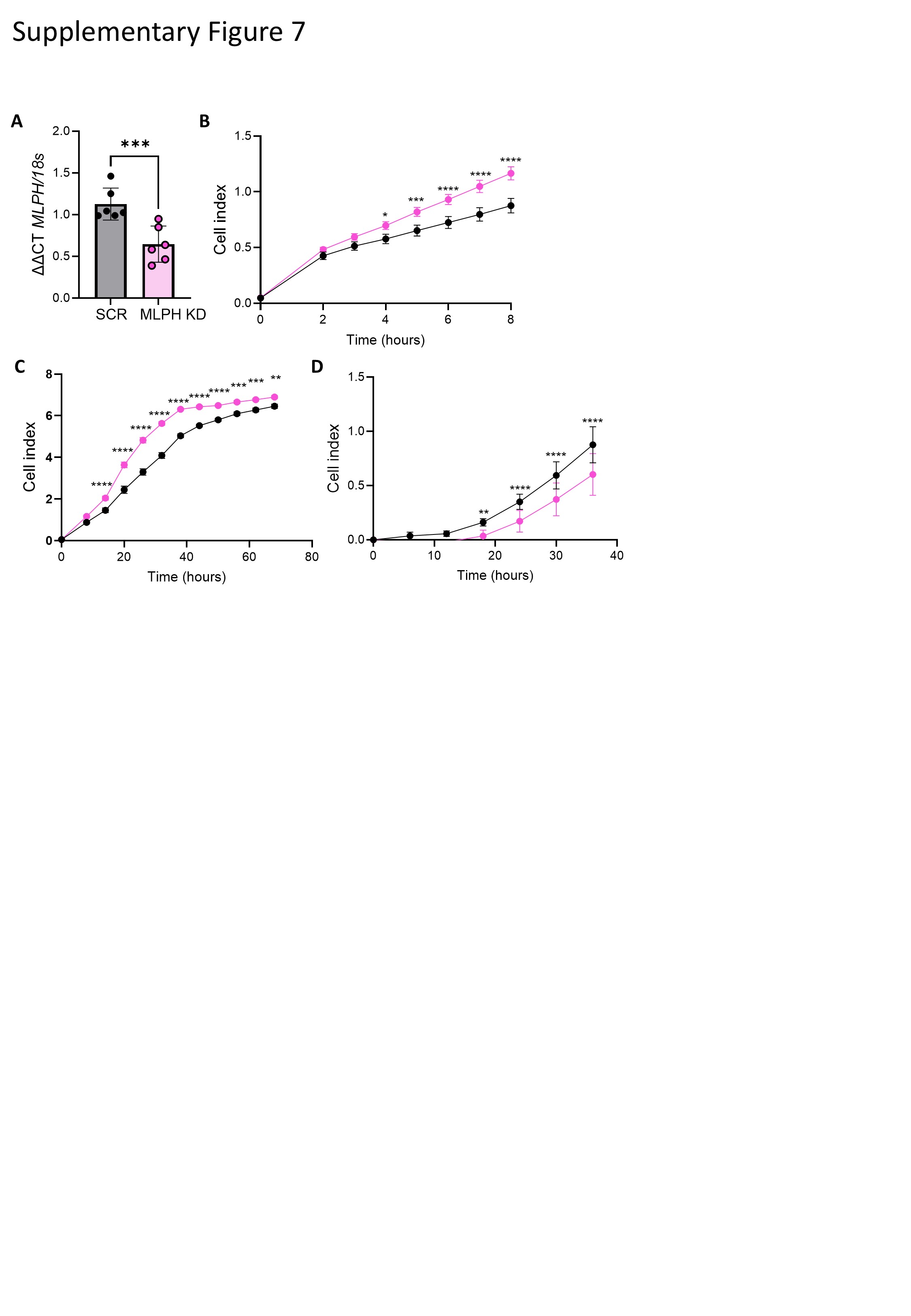


Figure S7. MLPH was required for extravillous trophoblast (EVT) invasion. A. MLPH production in EVT was reduced by knockdown (KD; SCR, scramble siRNA, MLPH, MLPH siRNA). Lower MLPH had no effect on EVT adhesion (B) but significantly enhanced EVT proliferation (C) and impaired EVT invasion (D). Data shows mean+sem; *, p<0.05; **, p<0.01; ***, p<0.001; ****, p<0.0001; Statistical tests: A, paired t-test; B-D, two-way ANOVA. Figure created using GraphPad Prism.


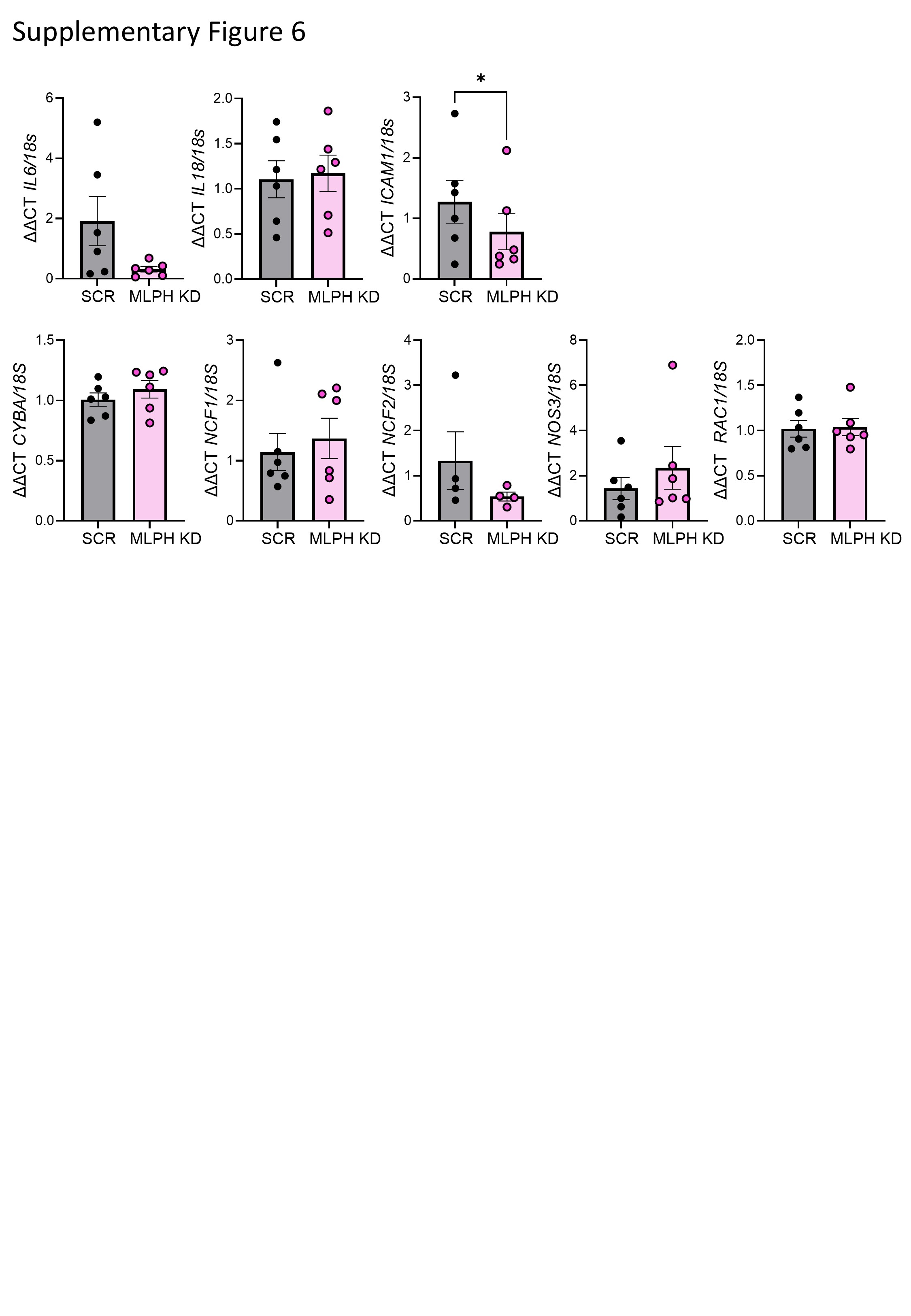


Figure S6. Gene expression in syncytialized cytotrophoblast progenitor cells (hTCP). MLPH knockdown (KD; SCR, scramble siRNA, MLPH, MLPH siRNA) reduced ICAM1 production in syncytializing hCTP. Data shows mean+sem; *, p<0.05; Statistical test: paired t-test. Figure created using GraphPad Prism.


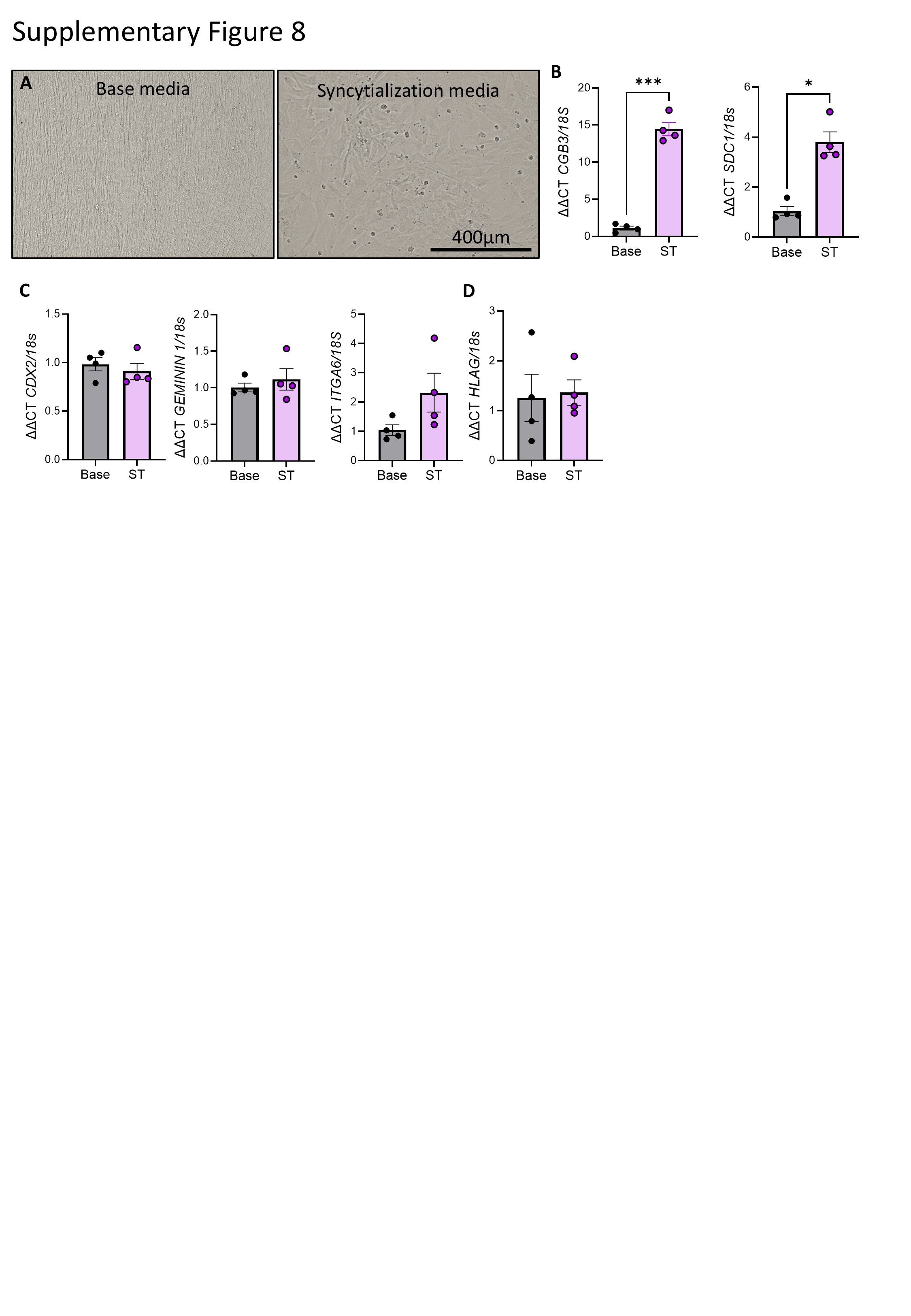


Figure S8. In vitro syncytialization of cytotrophoblast progenitor cells (hCTP). Treatment of hTCPs with syncytialization media (ST) A. altered cellular structure with multi-nucleated cells visible in culture and B. significantly increased production of syncytialization marker genes (*CGB3, SDC1*) but had no effect on C. cytrotrophoblast marker genes (*CDX2, GEMININ, ITGA6*) or D. extravillous trophoblast marker genes (*HLAG*). Data shows mean+sem; *, p<0.05; ***, p<0.001; Statistical tests: B-D, paired t-test. B-D created using GraphPad Prism.
